# Advances and Perspectives in Curcumin Regulation of Systemic Metabolism: A Focus on Multi-Organ Mechanisms

**DOI:** 10.3390/antiox15010109

**Published:** 2026-01-14

**Authors:** Dingya Sun, Jialu Wang, Xin Li, Jun Peng, Shan Wang

**Affiliations:** 1 Department of Pharmacology, Xiangya School of Pharmaceutical Sciences, Central South University, Changsha 410083, China; sundingya627@163.com; 2 Department of Pharmaceutical Engineering, College of Chemistry and Chemical Engineering, Central South University, Changsha 410083, China; m16680808529@163.com; 3 Hunan Provincial Key Laboratory of the Research and Development of Novel Pharmaceutical Preparations, College of Pharmacy, Changsha Medical University, Changsha 410219, China; tengyunxin2010@163.com

**Keywords:** curcumin, metabolic diseases, insulin resistance, gut microbiota, mitochondrial function, inflammation, oxidative stress

## Abstract

Curcumin, a natural polyphenol derived from turmeric, functions as a potent exogenous antioxidant and exhibits a range of benefits in the prevention and management of metabolic diseases. Despite its extremely low systemic bioavailability, curcumin demonstrates significant bioactivity in vivo, a phenomenon likely attributable to its accumulation in the intestines and subsequent modulation of systemic oxidative stress and inflammation. This article systematically reviews the comprehensive regulatory effects of curcumin on systemic metabolic networks—including glucose metabolism, amino acid metabolism, lipid metabolism, and mitochondrial metabolism—and explores their molecular basis, particularly how curcumin facilitates systemic metabolic improvements by alleviating oxidative stress and interacting with inflammation. Preclinical studies indicate that curcumin accumulates in the intestines, where it remodels the microbiota through prebiotic effects, enhances barrier integrity, and reduces endotoxin influx—all of which are critical drivers of systemic oxidative stress and inflammation. Consequently, curcumin improves insulin resistance, hyperglycemia, and dyslipidemia across multiple organs (liver, muscle, adipose) by activating antioxidant defense systems (e.g., Nrf2), enhancing mitochondrial respiratory function (via PGC-1α/AMPK), and suppressing pro-inflammatory pathways (e.g., NF-κB). Clinical trials have corroborated these effects, demonstrating that curcumin supplementation significantly enhances glycemic control, lipid profiles, adipokine levels, and markers of oxidative stress and inflammation in patients with obesity, type 2 diabetes, and non-alcoholic fatty liver disease. Therefore, curcumin emerges as a promising multi-target therapeutic agent against metabolic diseases through its systemic antioxidant and anti-inflammatory networks. Future research should prioritize addressing its bioavailability limitations and validating its efficacy through large-scale trials to translate this natural antioxidant into a precision medicine strategy for metabolic disorders.

## 1. Introduction

Curcumin ((1E,6E)-1,7-bis(4-hydroxy-3-methoxyphenyl)hepta-1,6-diene-3,5-dione) is a natural lipophilic polyphenolic compound extracted from the rhizomes of turmeric. As an active ingredient utilized in traditional Chinese and Indian medicine for centuries, its modern pharmacological value has been progressively unveiled since its isolation in 1815. Studies have demonstrated that curcumin exhibits a broad spectrum of biological activities, including antioxidant, anti-inflammatory, antibacterial, antitumor, and neuroprotective effects, by modulating multiple targets such as protein kinases, growth factors, transcription factors, and chemokines [1]. As a dietary supplement recognized by the U.S. FDA as “Generally Recognized As Safe” (GRAS) [2], curcumin has attracted significant attention in the field of metabolic disease research in recent years due to its capacity to maintain energy homeostasis by targeting key nodes in metabolism.

Metabolic disorders, characterized by abnormal glucose and lipid metabolism in various tissues and organs, pose a significant global public health challenge. These disorders are closely linked to energy surplus resulting from high-calorie diets and the prevalence of obesity, which directly contribute to the development of conditions such as metabolic syndrome, insulin resistance (IR), non-alcoholic fatty liver disease (NAFLD), and type 2 diabetes mellitus (T2DM) [3,4]. Taking T2DM as a case in point, its typical pathological features encompass excessive hepatic glucose production, impaired insulin secretion, and reduced insulin sensitivity across multiple tissues and organs [5,6]. Current treatment strategies primarily emphasize single-target interventions, which often struggle to effectively address the intricate network of metabolic diseases.

Curcumin has demonstrated significant potential in the prevention and treatment of obesity, IR, T2DM, and metabolic syndrome. Despite its extremely low oral bioavailability, extensive research has confirmed that its systemic beneficial effects are not solely dependent on the absorption of the prototype drug; rather, they arise from a ‘multi-node, multi-organ’ systemic regulatory mode [1]. Clinical metabolomics analysis provides direct evidence for this assertion: a randomized controlled trial revealed that curcumin intervention can significantly regulate numerous key metabolites in patients related to amino acid metabolism, the tricarboxylic acid (TCA) cycle, bile acid metabolism, and gut microbiota [7], thereby systematically confirming its potent ability to remodel the systemic metabolic network. This regulation is based on several key aspects: first, after oral administration, curcumin preferentially accumulates in the intestine, where it significantly influences host metabolism through bidirectional interactions with the gut microbiota. Specifically, gut microbes metabolize curcumin into active compounds, while curcumin reshapes microbial composition. For instance, it can be metabolized by NADPH-dependent enzymes within the gut microbiota into active metabolites such as dihydrocurcumin and tetrahydrocurcumin (THC) [8]. These metabolites not only retain but often enhance the anti-inflammatory and antioxidant activities of the parent compound, serving as crucial molecular mediators of its systemic effects [8]. Furthermore, curcumin selectively promotes the proliferation of beneficial bacteria such as *Akkermansia muciniphila*, *Lactobacillus*, and *Bifidobacterium*, while inhibiting the growth of opportunistic pathogens [9,10,11]. This process optimizes the gut microbiota structure, enhances intestinal barrier function, and increases short-chain fatty acid production. Thus, this bidirectional ‘metabolism-remodeling’ loop constitutes the microbiota-dependent mechanism underlying curcumin’s ‘low absorption, high activity’ properties, serving as a crucial foundation for its cross-organ metabolic regulatory effects [12]. Second, curcumin directly regulates glucose and lipid metabolism in peripheral tissues, including the liver, muscle, and adipose tissue, by activating essential signaling pathways such as AMP-activated protein kinase (AMPK) [7,13]. Third, it enhances cellular energy metabolism efficiency by promoting mitochondrial biogenesis and improving respiratory function [14]. Finally, curcumin mitigates the primary drivers of IR through its potent anti-inflammatory and antioxidant properties [15]. Furthermore, curcumin’s influence on amino acid and protein metabolism offers new insights into addressing metabolic complications, such as sarcopenia [16].

In current clinical applications and research, curcumin is widely utilized in various formulations, which primarily include traditional powders, standardized extracts, phospholipid complexes (such as Meriva^®^), nanoformulations (including nanoparticles, liposomes, and micelles), and combinations with bioavailability enhancers like piperine (e.g., Bioperine^®^) [17,18,19]. Commercially available products encompass a broad dosage range, from 80 mg/day of nano-curcumin to 2000 mg of standardized extracts [20,21]. In the studies covered by this review, curcumin was administered in various forms, including conventional powder, nanocapsules, and phospholipid complexes, with doses ranging from 80 to 2000 mg/day, depending on the disease model and formulation type. These variations in dosage forms and amounts directly impact its bioavailability and efficacy, serving as critical factors that necessitate systematic evaluation during its transition from a dietary supplement to a clinical treatment strategy.

This article systematically reviews the latest research advances regarding curcumin’s regulation of systemic metabolic functions, positioning its antioxidant and anti-inflammatory properties as central mechanisms underlying its multi-organ benefits. It provides an in-depth analysis of how curcumin enhances glucose homeostasis, lipid metabolism, and insulin sensitivity, while examining its role in coordinating systemic reductions in oxidative stress and inflammation from its intestinal site of action. By providing insights into gut microbiota modulation, enhanced mitochondrial respiration, and cellular signaling pathways, we underscore curcumin’s unique value as a multi-target therapeutic agent and discuss the challenges and future directions for its transition from a traditional natural product to clinical applications in metabolic disease management.

## 2. Methods

This study employed a narrative literature review approach, which involved the integration of relevant evidence through three distinct stages: systematic literature search, literature screening and inclusion, and evidence synthesis and narrative analysis.

A systematic literature search was conducted from October 2024 to October 2025 to comprehensively collect relevant studies up to October 2025. The search was performed in the PubMed and Web of Science databases, utilizing key search terms including curcumin, glucose metabolism, lipid metabolism, mitochondrial function, gut microbiota, insulin resistance, T2DM, obesity, NAFLD, and their related term variants. To ensure comprehensive coverage of curcumin’s multidimensional metabolic research, we conducted paired searches of core terms, such as ‘curcumin’ with ‘insulin resistance,’ ‘T2DM,’ and ‘NAFLD,’ among others. Additionally, we manually screened the reference lists of the included review articles to supplement important literature that may have been overlooked by electronic searches.

Subsequently, literature screening and inclusion were conducted according to predefined criteria. The types of studies included encompassed in vitro cell experiments, animal model studies, epidemiological investigations, and human clinical trials. The publication timeframe spanned from 1990 to October 2025. This study aimed to provide a comprehensive review of relevant mechanisms and evidence; therefore, no strict restrictions were imposed on study designs. However, particular emphasis was placed on including studies that elucidate molecular pathways or provide clinical efficacy data, with only published and full-text accessible articles considered. The screening process involved an initial selection based on titles and abstracts, followed by a full-text evaluation of potentially relevant studies.

A comprehensive narrative analysis was conducted on the included evidence. Due to the high heterogeneity among the included studies regarding experimental design, model systems, and outcome measures, this study did not perform a quantitative meta-analysis; instead, it adopted a narrative synthesis approach for qualitative integration. The information extracted from each study encompassed the authors, publication year, research model, curcumin intervention protocol, main findings, and associated molecular mechanisms. Based on the core scientific questions, the evidence was synthesized into several thematic discussions, including the pharmacokinetic characteristics of curcumin, its regulation of systemic and organ-specific glucose and lipid metabolism, effects on amino acid/protein metabolism and muscle function, modulation of mitochondrial function across multiple organs, mechanisms of influencing metabolism through gut microbiota and barrier function, and evidence for improving insulin resistance via anti-inflammatory and antioxidant effects. The research findings and discussions are systematically presented in subsequent sections, along with a summary of the clinical evidence for curcumin’s application in metabolic disorders.

## 3. Pharmacokinetics of Curcumin

The primary obstacle hindering the widespread clinical application of curcumin is its extremely low oral bioavailability, which is characterized by poor water solubility, a low gastrointestinal absorption rate, rapid metabolic clearance, and a significant first-pass effect [22,23].

Studies have demonstrated that following oral administration, curcumin preferentially distributes and accumulates in the intestines, with blood concentrations peaking within 1 to 2 h and gradually decreasing over the following 12 h [24]. Animal experiments indicate that curcumin predominantly accumulates in the gastrointestinal tract after oral intake. For instance, 30 min after rats ingested 400 mg of curcumin, 90% was found in the stomach and small intestine, while only trace amounts (less than 5 μg/mL) remained in the portal vein blood after 24 h. After this period, 38% of the ingested curcumin was retained in the cecum and large intestine [25]. When rats received a dose of 1000 mg/kg, nearly 75% of the curcumin was excreted in feces, with very low levels detected in urine [26]. In another study, after oral administration of curcumin at a dose of 0.1 g/kg in mice, the peak plasma concentration of free curcumin reached only 2.25 μg/mL within 15 min [27]. One hour post-administration, the organ concentrations displayed a gradient distribution (intestine 177.04 μg/g > spleen 26.06 μg/g ≈ liver 26.90 μg/g > kidney 7.51 μg/g > brain 0.41 μg/g), with only trace amounts present in the brain [27] (In the animal studies cited in this section, the term ‘curcumin’ typically denotes its pure form, specifically standard curcumin powder or extract, unless stated otherwise.). Curcumin, when administered via intravenous or intraperitoneal injection, is primarily metabolized in vivo into glucuronide conjugates, including tetrahydrocurcumin glucuronoside, hexahydrocurcumin glucuronoside, dihydrocurcumin glucuronoside, and curcumin glucuronoside itself. These conjugates represent the principal circulating and excretory forms of curcumin within bodily fluids, organs, and cells, with biliary excretion serving as the primary elimination pathway [27,28].

Human trial data further corroborate the absorption limitations of curcumin. Clinical trials indicate that after healthy subjects ingested 10 g of curcumin, only curcumin glucuronide/sulfate conjugates were detectable in the blood, with peak plasma concentrations ranging from 0.71 to 7.04 μg/mL and a half-life of 3.19 to 14.46 h. Free curcumin was nearly undetectable [29]. One hour following the oral administration of a 3.6 g dose of curcumin, the peak plasma concentration was measured at a low 11.1 nmol/L [30]. Similarly, after administering doses ranging from 4000 mg to 8000 mg per day, the mean peak serum concentration was only between 0.51 and 1.77 μM, with no curcumin detected in the urine [24]. Even at an oral dose of 8 g/day, intact curcumin remains barely detectable in plasma, with peak plasma concentrations only reaching 22 to 41 ng/mL [31]. Dose-escalation studies (500 mg to 12,000 mg) revealed that trace amounts of curcumin were only detected in subjects who consumed higher doses of 10,000 mg and 12,000 mg [32].

To address this limitation, various delivery systems—such as liposomes, polymeric micelles, nanoemulsions, and biopolymer nanoparticles—have been developed [18]. However, despite the implementation of these advanced drug delivery systems, the plasma concentration of curcumin typically remains below the effective in vitro dose of 5 μM [17,19]. As a result, most animal and human studies conducted to date still necessitate high-dose curcumin administration protocols [21,33,34,35]. Furthermore, the clinical dosage of curcumin must be tailored to accommodate different disease types [15,34,35]; for instance, a dosage ranging from 90 to 2000 mg/day has demonstrated significant therapeutic effects on oxidative stress and inflammation in patients [20,21,36,37]. It is important to note that the bioavailability of fresh or powdered curcumin within food matrices is higher compared to that of supplements, potentially due to the synergistic effects of curcuminoids or matrix interactions [38].

As previously noted, curcumin preferentially accumulates in the gastrointestinal tract following oral or intraperitoneal administration [23,25]. This accumulation allows curcumin to directly modulate the abundance, diversity, and composition of the gut microbiota, thereby influencing intestinal barrier function and associated signaling pathways [12,39,40]. Specific mechanisms will be elaborated upon in subsequent chapters.

It is noteworthy that the gut microbiota can not only be regulated by curcumin but also transform it into various active metabolites, thereby generating multiple pharmacological activities. Consequently, the gut microbiota modulated by curcumin can, in turn, influence the absorption, metabolism, and overall therapeutic potential of curcumin, demonstrating its prebiotic properties [9,41]. Studies have confirmed that only 5–10% of the prototype curcumin is absorbed by the small intestine after ingestion, while 90–95% is metabolized by the gut microbiota into absorbable active products [42]. This indicates that the gut microbiota is a key regulator determining the pharmacological activity of curcumin, providing a basis for explaining the paradox between its low bioavailability and extensive pharmacological benefits, as well as significant clinical effects [9,12,39,41]. Notably, conventional assays often overlook the analysis of curcumin derivatives, potentially leading to an underestimation of the absorption level of curcumin.

In summary, the low systemic bioavailability of curcumin represents a significant challenge for its clinical translation. To address this issue, various delivery strategies—including liposomes, nanoparticles, and phospholipid complexes—have been developed and shown potential in enhancing plasma concentrations [18]. However, even with advanced delivery systems, the plasma levels achieved often remain below the effective concentrations observed in vitro, exhibiting considerable inter-individual variability [17,19]. This phenomenon has led researchers to reevaluate the paradigm of curcumin’s action: its broad-spectrum systemic metabolic benefits may not primarily depend on high circulating levels of the parent compound, but rather arise from its localized high-concentration accumulation in the intestinal tract, microbial transformation into active metabolites [12,39,41], and subsequent initiation of systemic regulation through the ‘gut-organ axis.’ Therefore, the low bioavailability of curcumin and its unique ‘multi-organ, multi-target’ mode of action constitute a seemingly paradoxical yet intrinsically unified characteristic. Understanding and leveraging this property—such as by designing strategies targeting the gut or combining curcumin with prebiotics—rather than merely pursuing higher plasma concentrations, may offer more promising avenues for developing precision metabolic interventions based on curcumin. The following sections will systematically elucidate how curcumin regulates systemic metabolism through a multi-organ integrated network under this ‘low absorption, high activity’ paradigm.

## 4. Curcumin Regulation of Glucose Metabolism

### 4.1. Overall Improvement in Glucose Homeostasis by Curcumin

Elevated fasting blood glucose levels are primarily driven by enhanced hepatic gluconeogenesis, which reflects impaired glucose regulation within the body. Increased hepatic glucose production (HGP) is a significant contributor to fasting hyperglycemia in patients with type 2 diabetes [43,44]. Specifically, fasting blood glucose levels arise from endogenous glucose production, and pyruvate, a key substrate for gluconeogenesis, serves as an indicator of hepatic gluconeogenic capacity through its tolerance assessment. Studies have demonstrated that curcumin can reverse abnormal pyruvate tolerance in mice subjected to a high-fat diet (HFD) and mitigate hyperglycemic responses induced by glucagon in healthy mice. This indicates curcumin’s potential effectiveness in alleviating hyperglycemia by inhibiting hepatic gluconeogenesis [45,46]. Curcumin significantly reduces hyperglycemia and glycated hemoglobin (HbA1C) levels in diabetic animal models induced by an HFD by inhibiting the activity of key rate-limiting enzymes in hepatic gluconeogenesis, specifically glucose-6-phosphatase (G6Pase) and phosphoenolpyruvate carboxykinase (PEPCK). Additionally, curcumin enhances hepatic glucokinase activity, thereby improving glucose tolerance and insulin sensitivity [47,48,49]. Clinical evidence further supports its hypoglycemic effects; for instance, a double-blind randomized trial demonstrated that type 2 diabetes patients who took 80 mg of nano-curcumin capsules for three consecutive months experienced significant reductions in fasting blood glucose and HbA1C levels [50]. Furthermore, multiple trials have confirmed its efficacy in treating patients with T2DM [5,51] (Table 1).

Insulin resistance is a key factor contributing to elevated blood glucose levels in patients with obesity and T2DM. Improving IR can significantly delay disease progression. Clinical studies have demonstrated that curcumin effectively reduces fasting blood glucose, HbA1c, and the IR index in overweight and obese patients with T2DM [5,6,52], while also enhancing glucose and lipid metabolism and insulin sensitivity [6]. Notably, curcumin does not exhibit a significant effect on fasting blood glucose levels in healthy individuals [53]. Numerous studies have confirmed curcumin’s regulatory effect on IR [52,54,55], and meta-analyses further illustrate that curcumin can systematically lower fasting blood glucose, HbA1c, and homeostatic model assessment of insulin resistance (HOMA-IR) in patients with metabolic diseases [56,57]. These findings underscore the substantial clinical value of curcumin in improving glucose homeostasis and mitigating IR.

### 4.2. Core Mechanisms Regulating Hepatic Glucose Metabolism

**Figure 1:**
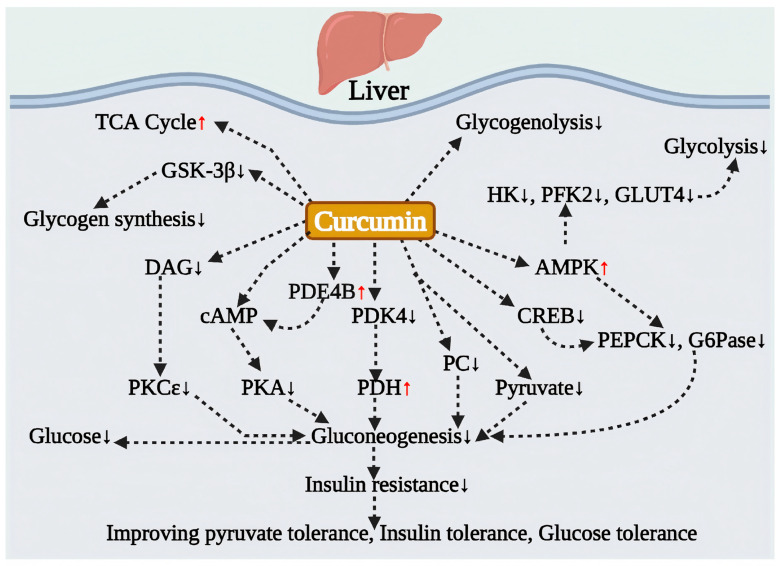
Curcumin regulates hepatic glucose metabolism. Curcumin inhibits hepatic gluconeogenesis by activating the AMPK signaling pathway and suppressing key enzymes such as G6Pase and PEPCK, while simultaneously promoting glycogen synthesis. Additionally, it modulates the interplay between glucose and lipid metabolism by reducing cAMP/PKA signaling and fatty acid oxidation, thereby enhancing hepatic insulin sensitivity. Colored arrows indicate the inhibitory (black) or stimulatory (red) effects of curcumin on downstream signaling targets, finally involved in regulating a variety of intracellular physiological processes. (Original drawing, created with BioRender, https://biorender.com, accessed on 8 January 2026).

Curcumin profoundly regulates glucose metabolism by targeting critical steps in hepatic gluconeogenesis and glycogen synthesis (Figure 1). A central mechanism of its action is the activation of the AMPK signaling pathway. Studies have demonstrated that curcumin reduces glucose production in primary hepatocytes in vitro through an insulin-independent mechanism, which involves the activation of AMPK and the inhibition of key rate-limiting enzymes for gluconeogenesis, specifically G6Pase and PEPCK. This process significantly decreases both gluconeogenesis and glycogenolysis [58]. Furthermore, this effect has been substantiated in whole-animal experiments: curcumin can reverse abnormal pyruvate tolerance—pyruvate being a key substrate for gluconeogenesis—in mice fed an HFD and alleviate hyperglycemia induced by glucagon, thereby confirming its potent ability to inhibit hepatic glucose output [45,46].

While effectively suppressing gluconeogenesis, curcumin also promotes hepatic glycogen synthesis, thereby bidirectionally regulating glucose balance in the liver. In rat models of obesity and type 2 diabetes, curcumin treatment significantly increased hepatic glycogen levels that had been reduced due to abnormal blood sugar [59]. Further mechanistic studies revealed that curcumin could enhance hepatic glycogen synthesis in fasting mice by inhibiting the expression of glycogen synthase kinase (GSK3-β) [59]. Collectively, these findings indicate that through the activation of AMPK signaling, curcumin can simultaneously inhibit hepatic glucose production and promote hepatic glycogen storage, thereby playing a central role in regulating glucose homeostasis at the hepatic level (Figure 1).

### 4.3. Improving Hepatic Glucose Metabolism by Intervening in Glucose-Lipid Metabolic Crosstalk

The imbalance between hepatic gluconeogenesis and lipid metabolism disorders is intricately intertwined, collectively promoting the progression of metabolic diseases [60]. Research indicates that curcumin intervenes in this complex pathological process through multiple mechanisms (Figure 1). Firstly, in palmitic acid-stimulated primary hepatocytes, curcumin reverses the glucagon-mediated activation of the cAMP/PKA pathway, thereby suppressing endogenous glucose production. This suggests that modulating cAMP homeostasis may serve as an upstream target for inhibiting gluconeogenesis [46]. In an HFD mouse model, curcumin maintains hepatic PDE4B activity (which is responsible for cAMP degradation) in an AMPK-dependent manner, reduces cAMP accumulation, and blocks PKA activation. Additionally, it suppresses the expression of PGC-1α and its downstream gluconeogenic enzymes, PEPCK and G6Pase, thereby decreasing hepatic glucose production (HGP) through the inactivation of CREB via dephosphorylation [46].

Curcumin plays a significant role in the gluconeogenesis pathway driven by acetyl-CoA derived from fatty acid oxidation. It reduces the accumulation of acetyl-CoA and its activating effect on pyruvate carboxylase (PC) by inhibiting the expression of pyruvate dehydrogenase kinase 4 (PDK4) and preserving the activity of pyruvate dehydrogenase (PDH). This action effectively obstructs the hepatic gluconeogenesis process that is initiated by pyruvate carboxylation. Additionally, this mechanism is linked to a reduction in hepatic fatty acid oxidation and a decrease in the activity of mitochondrial complex I [46]. It is important to note that excessive free fatty acids (FFAs) in circulation serve not only as critical substrates for stimulating hepatic glucose production but also contribute to the worsening of systemic IR. Curcumin plays a crucial role in this process. In an HFD-induced obese mouse model, treatment with curcumin not only improved glucose tolerance but also reduced circulating free fatty acid (FFA) levels by inhibiting the breakdown of adipose tissue [46]. Additionally, by downregulating the expression of the fatty acid transporter protein CD36 on hepatocyte membranes, curcumin blocked the intrahepatic transport of FFAs, thereby alleviating ectopic lipid deposition in the liver [45]. Furthermore, curcumin restored the normal inhibitory effect of insulin on hepatic gluconeogenesis by reducing hepatic diacylglycerol (DAG) deposition and inhibiting the translocation of protein kinase C epsilon (PKCε), thereby enhancing hepatic insulin sensitivity at multiple levels [45]. Notably, the association between DAG deposition in muscle tissue and IR has also been observed in patients with obesity and T2DM [61].

Moreover, the beneficial effects of curcumin extend to the process of hepatic fibrosis, which is closely linked to glycolipid metabolic disorders. Activated hepatic stellate cells (HSCs) play a central role in hepatic fibrosis and rely heavily on glycolytic metabolism to sustain their activated phenotype. Research has demonstrated that curcumin activates the AMPK signaling pathway, which targets and inhibits the expression of hexokinase (HK), 6-phosphofructo-2-kinase (PFK2), and glucose transporter (GLUT4) in HSCs. This inhibition of glycolytic metabolism leads to a reduction in intracellular glucose levels and lactate production, ultimately contributing to its anti-fibrotic effects [62].

From a broader perspective of systemic energy metabolism, curcumin exhibits a remarkable capacity for remodeling. Under pathological conditions such as hyperlipidemia, the activation of glycolysis and gluconeogenesis results in an excessive accumulation of acetyl-CoA, which inhibits the TCA cycle and forces energy metabolism to shift towards lipid oxidation. Metabolomic analyses of mouse models on an HFD have shown that curcumin treatment significantly increases the levels of TCA cycle metabolites, including citrate, succinate, and cis-aconitate, while simultaneously reducing the concentrations of pyruvate, lactate, and glucose. This confirms curcumin’s ability to inhibit abnormal glycolysis and gluconeogenesis pathways, effectively redirecting energy metabolism towards a normal oxidative metabolic pattern [63]. Clinical trials have demonstrated that curcumin significantly reduces the levels of TCA cycle intermediates in patients’ serum, including succinate, citrate, and α-ketoglutarate. This reduction indicates curcumin’s ability to optimize TCA cycle function and fundamentally improve glucose metabolism disorders [7]. Collectively, these findings illustrate that curcumin, by regulating core metabolic nodes in the liver, exerts systemic beneficial effects by integrating the glycolipid metabolic network and reshaping whole-body energy homeostasis (Figure 1).

### 4.4. Curcumin Regulation and Improvement in Muscle Metabolic Function

Skeletal muscle plays a crucial role in glucose metabolism within the body, and its dysfunction can directly contribute to systemic IR. In animal models of obesity and diabetes, the expression levels of the glucose transporter GLUT4 and glycogen content in skeletal muscle are significantly diminished [59,64,65,66]. Similarly, in the brain tissues of animals with diabetes induced by an HFD or streptozotocin (STZ), the expression of GLUT1 and GLUT3 also exhibits a downward trend, which is a characteristic pathological feature of chronic hyperglycemia [67,68]. This suppression of glucose transporter expression is regarded as an adaptive protective mechanism by the body in response to persistent hyperglycemia, as it limits excessive glucose influx to prevent glucotoxicity damage.

**Figure 2:**
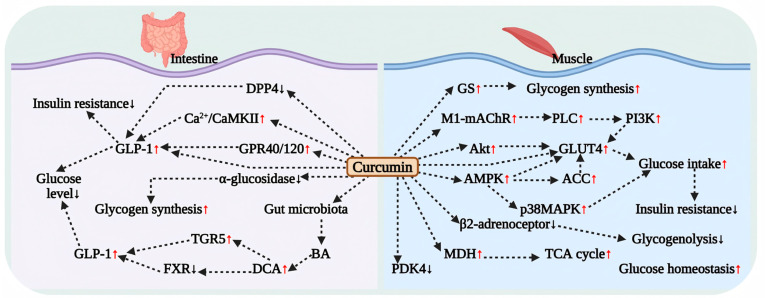
Curcumin regulates glucose metabolism in the intestine and muscle. In the intestines, curcumin regulates gut-derived hormones and delays sugar absorption by promoting the secretion of GLP-1 and inhibiting α-glucosidase, among other mechanisms. In the muscles, curcumin enhances glucose utilization by activating the AMPK/Akt signaling pathway, thereby promoting glucose uptake and glycogen synthesis. Colored arrows indicate the inhibitory (black) or stimulatory (red) effects of curcumin on downstream signaling targets, finally involved in regulating a variety of intracellular physiological processes. (Original drawing, created with BioRender, https://biorender.com, accessed on 8 January 2026).

Curcumin enhances glucose uptake by activating the AMPK and Akt signaling pathways (Figure 2). In C2C12 myocytes, curcumin stimulates AMPK-ACC and Akt signaling, promoting GLUT4 translocation to the plasma membrane and thereby facilitating glucose uptake [69]. Na et al. demonstrated that curcumin reverses palmitate-induced impairment of glucose uptake in L6 myotubes in a dose-dependent manner, a mechanism that involves AMPK-mediated upregulation of GLUT4 membrane expression [70]. Furthermore, Deng et al. found that curcumin improves insulin sensitivity by restoring IRS-1 expression, stimulating Akt phosphorylation, and activating MAPK signaling in palmitate-treated C2C12 cells [71]. Additional animal studies have confirmed that curcumin activates the AMPK-p38MAPK signaling pathway, promoting glucose uptake in mouse skeletal muscle [71].

Furthermore, curcumin has been shown to regulate muscle glucose metabolism through various mechanisms. Cheng et al. reported that curcumin enhances GLUT4 membrane localization in rat skeletal muscle by activating the M1-mAChR-PLC-PI3K pathway, which subsequently promotes glucose uptake [72]. In diabetic animal models, curcumin treatment upregulates GLUT4 expression in skeletal muscle, leading to significant improvements in blood glucose levels and body weight [59]. Additionally, curcumin treatment has been observed to increase GLUT4 expression in white adipose tissue in obese mice [12]. Moreover, curcumin effectively maintains glucose homeostasis by inhibiting β(2)-adrenergic receptor-mediated glycogenolysis and restoring mitochondrial malate dehydrogenase (MDH) activity in the skeletal muscle of diabetic rats [73,74].

Curcumin also exerts regulatory effects on glycogen synthesis and PDK4 expression. In diabetic rat skeletal muscle and insulin-resistant L6 myotube cells, curcumin significantly enhances glucose metabolic activity in skeletal muscle by inhibiting PDK4 expression and reducing the phosphorylation level of glycogen synthase (GS), thereby promoting glycogen synthesis. Interestingly, Lakshmanan et al. found that curcumin treatment reversed the compensatory elevation of GLUT4 expression in the brains of diabetic encephalopathy rats. This may be related to the inhibition of AMPK activation-mediated enhancement of GLUT4 expression. However, the significant reduction in blood glucose levels after treatment fully confirms the overall hypoglycemic efficacy of curcumin [70,75]. In summary, curcumin exerts its hypoglycemic effect by regulating muscle glucose metabolism activity through multiple targets, although the detailed mechanisms still require further elucidation (Figure 2).

### 4.5. Effect of Curcumin on Intestinal Glucose Metabolism

Curcumin enhances GLP-1 secretion through various pathways, thereby improving glucose metabolism. In turn, GLP-1 synergistically alleviates hyperglycemia and IR by increasing the glucose sensitivity of pancreatic β cells, promoting insulin secretion, and inhibiting glucagon release (Figure 2).

In both cellular and animal models, curcumin has been demonstrated to directly stimulate the secretion of GLP-1 and elucidate its underlying mechanisms. Studies indicate that curcumin enhances GLP-1 secretion in a dose-dependent manner in mouse STC-1 cells, which are representative of enteroendocrine L cells [13]. Takikawa et al. confirmed that curcumin also promotes GLP-1 secretion in the mouse enteroendocrine L cell line (GLUTag cells) by activating the Ca^2+^/CaMKII signaling pathway [76,77]. In animal models, curcumin has been shown to increase plasma GLP-1 and insulin levels in rats with impaired glucose tolerance by activating GPR40/120 receptors, leading to significant improvements in glucose tolerance and blood sugar control [77]. More importantly, in obesity models, curcumin restores the abundance of intestinal L-cells and promotes GLP-1 secretion through the gut microbiota-bile acid-TGR5/FXR axis, markedly ameliorating the obesity phenotype. This mechanism may be central to its regulation of GLP-1 levels [78]. Furthermore, randomized clinical trials have confirmed the enhancing effect of curcumin on human GLP-1 levels. Studies indicate that 24 weeks of curcumin supplementation significantly increases serum GLP-1 levels in patients [40], providing direct human data to support its clinical application (Table 1).

In addition to promoting GLP-1 secretion, curcumin may also regulate intestinal glucose metabolism by inhibiting the activity of related enzymes. In vitro studies have confirmed that curcumin can act as a potential inhibitor of α-glucosidase and dipeptidyl peptidase-4 (DPP-4, which causes rapid degradation and inactivation of endogenous GLP-1). However, in vivo experiments indicate that its enzyme inhibitory effects may differ due to bioavailability limitations [79,80,81]. In a mouse model of obesity induced by a high-fat, high-sugar diet, curcumin achieved blood glucose control by inhibiting small intestinal α-glucosidase activity and promoting glycogen synthesis in the liver and muscles. Nevertheless, its inhibitory effect on DPP-4 was limited [81], potentially related to its low bioavailability characteristics in vivo [82]. In summary, curcumin regulates intestinal glucose metabolism through a synergistic multi-pathway strategy that primarily promotes GLP-1 secretion while secondarily inhibiting intestinal sugar-digesting enzymes, presenting a potential therapeutic strategy for blood glucose management in obese and type 2 diabetic patients (Figure 2).

## 5. Curcumin Regulates Lipid Metabolism

### 5.1. Effect of Curcumin on Blood Lipid Metabolism

Blood lipid levels, including total cholesterol (TC), low-density lipoprotein cholesterol (LDL-C), high-density lipoprotein cholesterol (HDL-C), and triglycerides (TG), serve as fundamental indicators for evaluating metabolic health in humans. Disorders in lipid metabolism are notably prevalent among individuals with obesity and related metabolic diseases. Clinical studies have demonstrated that curcumin can enhance lipid metabolic homeostasis by targeting multiple pathways, leading to a significant reduction in dyslipidemia levels in patients suffering from obesity, T2DM, NAFLD, and metabolic syndrome (Table 1).

In populations characterized by obesity and overweight, several randomized controlled trials have substantiated the lipid-modulating effects of curcumin (Table 1). One study demonstrated that overweight or obese patients with NAFLD who consumed nanocapsules of curcumin (80 mg/day) for three consecutive months experienced significant reductions in fasting blood glucose (FBG), glycated hemoglobin (HbA1C), insulin resistance index (HOMA-IR), TG, TC, and LDL-C levels. In contrast, HDL-C and the quantitative insulin sensitivity index (QUICKI) showed marked improvements [83]. Another randomized controlled trial indicated that continuous intake of 1 g/day of curcumin for 30 days led to a significant decrease in plasma TG levels among obese patients [84]. Furthermore, after 8 weeks of supplementation with plant-based curcumin tablets (800 mg/day), overweight subjects exhibited a significant reduction in plasma triglyceride (TG) and HDL-C levels. Additionally, notable improvements were observed from baseline in fasting plasma insulin (FPI), HOMA index, waist circumference, blood pressure, liver enzymes (transaminases and gamma-glutamyl transferase (γ-GT)), hepatic steatosis index, and serum cortisol levels [85].

Curcumin has been shown to have significant intervention effects in patients with T2DM, who frequently present with concurrent hyperlipidemia (Table 1). Clinical trials indicate that daily doses of curcumin at 1500 mg and 1000 mg can significantly reduce serum TG, lipoprotein(a) [LP(a)], and non-LDL cholesterol levels in T2DM patients, while simultaneously increasing HDL-C levels [86]. Additionally, another study demonstrated that a daily intake of 80 mg of nano-curcumin capsules over 12 weeks led to significant reductions in TG, TC, LDL-C, very-low-density lipoprotein cholesterol (VLDL-C), and the TC/HDL-C ratio [51]. The mechanism underlying these effects is partially attributed to the activation of lipoprotein lipase (LPL), as increased LPL activity enhances the hydrolysis of TG. Furthermore, a double-blind, placebo-controlled trial revealed that daily supplementation with 300 mg of curcumin for three months significantly reduced serum free fatty acids (FFAs) and TG levels in overweight and obese T2DM subjects, which was accompanied by an increase in LPL activity and an overall improvement in lipid metabolism disorders [51,52].

In patients with NAFLD, curcumin has been shown to exert beneficial effects on dyslipidemia (Table 1). For example, a study found that after NAFLD patients were administered 500 mg/d of curcumin for 8 consecutive weeks, their serum triglyceride (TG), TC, and LDL-C levels were significantly lower compared to those in the placebo group [87]. Furthermore, research conducted by Panahi et al. indicated that a dosage of 1000 mg/d of curcumin over 8 weeks led to a significant reduction in liver fat content, as well as levels of LDL-C, TG, TC, and non-high-density lipoprotein cholesterol (non-HDL-C) in NAFLD patients [88]. Additionally, dose–response studies have demonstrated that administering 250 mg/d of phosphatidyl curcumin capsules for 8 weeks significantly increased HDL-C levels in NAFLD patients [89]. Increasing the dosage to 1500 mg/d further reduced serum levels of TC, LDL-C, HDL-C, TG, and non-HDL-C while improving the hepatic lipid index [90].

It is noteworthy that the lipid-regulating effects of curcumin are associated with the modulation of adipokines. Adiponectin, a crucial factor that enhances insulin sensitivity and inhibits fatty acid synthase activity, plays a pivotal role in ameliorating insulin resistance and remodeling glucose and lipid metabolic homeostasis. Clinical studies have confirmed that curcumin intervention significantly elevates serum adiponectin levels while reducing leptin levels in patients, thereby synergistically improving symptoms of lipid metabolism disorders [54,89,91].

### 5.2. Curcumin Regulation of Hepatic Lipid Metabolism

#### 5.2.1. Core Regulatory Strategies: Inhibiting Lipogenesis and Promoting Lipolysis

**Figure 3:**
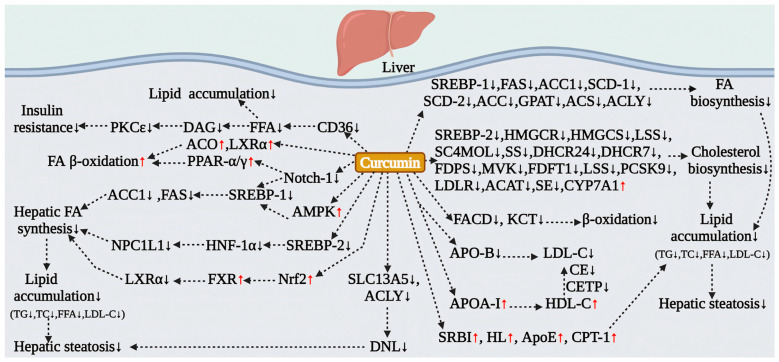
Curcumin regulates hepatic lipid metabolism. Curcumin reduces hepatic lipid accumulation by inhibiting lipogenesis through the AMPK/SREBP axis and promoting fatty acid oxidation. Additionally, it downregulates CD36-mediated fatty acid uptake and enhances cholesterol catabolism and bile acid synthesis. Colored arrows indicate the inhibitory (black) or stimulatory (red) effects of curcumin on downstream signaling targets, finally involved in regulating a variety of intracellular physiological processes. (Original drawing, created with BioRender, https://biorender.com, accessed on 8 January 2026).

The liver serves as the central regulator of lipid metabolism and is subject to functional disturbances that result in abnormal lipid accumulation. Curcumin has been shown to effectively reverse hepatic and circulatory lipid metabolic disorders induced by dietary obesity (Figure 3). In animal models of dietary obesity, curcumin significantly reduces hepatic lipid content, inhibits lipid droplet formation, and ameliorates steatosis [45,46,92]. This is achieved by suppressing the activities of fatty acid synthase (FAS), the rate-limiting enzyme for cholesterol synthesis (HMGCR), and acyl-CoA cholesterol acyltransferase (ACAT), while simultaneously activating hepatic fatty acid β-oxidation. This mechanism synergistically reduces hepatic lipid peroxidation by inhibiting fatty acid and cholesterol biosynthesis, as evidenced by significant reductions in hepatic cholesterol and triglyceride levels [92]. For instance, in an HFD mouse model, curcumin reduces the accumulation of acetyl-CoA (the end product of β-oxidation) in the liver by downregulating the expression of fatty acyl-CoA dehydrogenase (FACD) and 3-ketoacyl-CoA thiolase (KCT), thereby inhibiting fatty acid oxidation [46].

#### 5.2.2. Inhibiting Lipogenesis via the AMPK/SREBP Signaling Axis

Extensive research has confirmed that the activation of AMPK signaling represents the primary mechanism through which curcumin multi-dimensionally regulates hepatic lipid metabolism (Figure 3). Both pharmacological and clinical doses of curcumin significantly inhibit the activity of hepatic lipid synthesis genes induced by an HFD [47,93], thereby suppressing de novo fatty acid synthesis and lipid accumulation [94,95,96]. Studies conducted at the cellular level have demonstrated that in Huh-7/SRE-Luc cells, curcumin broadly inhibits the transcription of SREBP target genes, including nine fatty acid and triglyceride synthesis genes (such as SREBP-1, FAS, ACC1, and SCD-1) as well as eleven cholesterol synthesis-related genes (including SREBP-2, HMGCR, HMGCS, and MVK), resulting in a marked reduction in cholesterol and triglyceride levels [93]. Further investigations using animal models have revealed that in HFD-induced obese mice, curcumin downregulates the expression of hepatic cholesterol biosynthesis genes (such as HMGCR, FDPS, DHCR24) and fatty acid metabolism genes (including ACC1, FAS, and SCD-1) at the mRNA level, while simultaneously upregulating the expression of SRBI, HL, and ApoE to accelerate lipolysis. This indicates that curcumin reduces hepatic lipid synthesis and promotes decomposition by inhibiting the SREBP-1 and SREBP-2 pathways [93,95]. Additionally, curcumin effectively reverses HFD-induced hepatic steatosis by activating AMPK signaling and downregulating SREBP-1 along with its downstream target genes, ACC1 and FAS [94].

#### 5.2.3. Regulating Fatty Acid Transport and Alleviating Lipotoxicity

Curcumin effectively alleviates hepatic lipotoxicity by modulating the transport and metabolism of fatty acids. The fatty acid transporter CD36 plays a critical role in mediating hepatic lipid accumulation. Curcumin reduces circulating lipid levels and diminishes their transport into the liver by inhibiting the expression of CD36 in the liver of HFD-induced obese mouse models, consequently decreasing ectopic lipid deposition [45]. Additionally, curcumin activates AMPK to inhibit the cAMP/PKA signaling pathway in adipose tissue, thereby suppressing lipid breakdown in the adipose tissue of HFD-fed mice. This mechanism synergistically downregulates the expression of hepatic CD36, leading to a reduction in the influx of free fatty acids (FFAs) into the liver, which alleviates lipid deposition and insulin resistance [45]. Clinical trials have further validated the efficacy of curcumin in reducing serum TG, free fatty acids (FFAs), fasting blood glucose, and HbA1c levels in patients with non-alcoholic simple fatty liver (NASFL). These findings suggest that the inhibition of hepatic DNL may be a crucial mechanism through which curcumin ameliorates fatty liver disease [40] (Figure 3).

#### 5.2.4. Regulation of Lipid Metabolism via Multiple AMPK-Independent Pathways

Curcumin exerts lipid-regulating effects through several AMPK-independent pathways (Figure 3). In an HFD mouse model, curcumin enhances the expression of hepatic PPARα and LXRα, thereby activating the transcription of genes involved in fatty acid oxidation and cholesterol transport. This is evidenced by the upregulation of APOA-I and the downregulation of HMGR and APO-B, which significantly reduces hepatic cholesterol and triglyceride accumulation [97]. Additionally, by inhibiting the activity of cholesteryl ester transfer protein (CETP), curcumin prevents the transfer of cholesteryl esters and TG between HDL and LDL, reduces VLDL production, and enhances LDL clearance, ultimately leading to increased HDL-C levels [97]. Zhao et al. discovered that curcumin downregulates SREBP-1 expression by inhibiting the Notch-1 signaling pathway, which suppresses hepatic lipogenic genes (FAS, ACC) while promoting the expression of lipolytic genes (PPAR-α, PPAR-γ, CPT-1), thereby improving lipid metabolism in the livers of HFD-fed rats [98]. Curcumin has been shown to influence the metabolism of bile acids and the excretion of cholesterol. It promotes the catabolism of fatty acids in the liver of HFD rats by activating the rate-limiting enzyme of fatty acid β-oxidation, acyl-CoA oxidase (ACO), which subsequently reduces hepatic triglyceride (TG) and plasma very-low-density lipoprotein triglyceride (VLDL-TG) levels [99]. Further research conducted by Babu et al. indicates that curcumin enhances cholesterol catabolism by activating cholesterol 7α-hydroxylase (CYP7A1), leading to decreased circulating lipid levels [100]. Additionally, curcumin accelerates the conversion of cholesterol to bile acids by upregulating hepatic cholesterol 7α-hydroxylase (CYP7A1), which results in lower serum levels of TC, TG, and LDL-C, while increasing the excretion of TG and TC in feces [101]. Furthermore, curcumin downregulates the expression of Niemann-Pick C1-like 1 (NPC1L1) in both the intestine and liver by inhibiting the SREBP-2/HNF1α signaling pathway, thereby reducing the reabsorption of biliary cholesterol and promoting the excretion of neutral cholesterol in feces, effectively reversing hepatic cholesterol accumulation [102]. Clinical studies have also confirmed that curcumin intervention can lower serum levels of bile acids, including taurocholic acid, chenodeoxycholic acid, and lithocholic acid, thereby regulating bile acid metabolism [7].

#### 5.2.5. Validation in NAFLD and Diabetic Models

The beneficial effects of curcumin on hepatic lipid metabolism have been validated across various disease models, indicating its potential for clinical application. In diabetic models, animal studies demonstrate that curcumin significantly reduces plasma levels of free fatty acids (FFAs), TC, and TG by inhibiting the activities of hepatic fatty acid synthase (FAS), carnitine palmitoyltransferase (CPT), 3-hydroxy-3-methylglutaryl-CoA reductase (HMGCR), and acyl-CoA cholesterol acyltransferase (ACAT), while simultaneously decreasing β-oxidation activity [47]. In streptozotocin (STZ)-induced type 1 diabetes models, curcumin mitigates renal lipid accumulation by activating the renal AMPK signaling pathway, which suppresses the expression of sterol regulatory element-binding protein 1c (SREBP-1c) and its downstream genes, including adipose differentiation-related protein (ADRP), acetyl-CoA carboxylase (ACC), and FAS [103]. In NAFLD and non-alcoholic steatohepatitis (NASH) models, curcumin treatment significantly reduces hepatic levels of TC, TG, and non-esterified fatty acids (NEFAs), and inhibits the expression of SREBP-1c, FAS, and CD36 by activating the nuclear factor erythroid 2-related factor 2 (Nrf2)/farnesoid X receptor (FXR)/liver X receptor alpha (LXRα) signaling axis, thereby decreasing hepatic lipid droplet accumulation [104]. In vitro studies have shown that curcumin can inhibit the abnormal elevation of SLC13A5 and ATP citrate lyase (ACLY) induced by oleic acid/palmitic acid (OPA) in primary hepatocytes, thereby reducing intracellular triglyceride (TG) accumulation. Subsequent in vivo studies have confirmed that curcumin restores citrate homeostasis in hepatocytes by inhibiting the SLC13A5 and ACLY signaling pathways, which synergistically reduce de novo lipogenesis (DNL) and ameliorate steatosis [105]. These multifaceted mechanisms of action have been partially validated in clinical trials of curcumin treatment [7,88,90], suggesting that curcumin may be a promising strategy for improving lipid metabolism disorders associated with obesity, T2DM, and NAFLD (Table 1).

### 5.3. Curcumin Regulation of Adipocyte Lipid Metabolism

**Figure 4:**
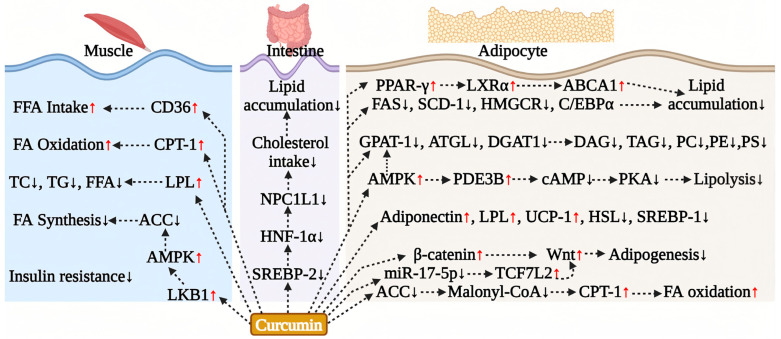
Curcumin regulates lipid metabolism in muscle, intestine, and adipose tissue. Curcumin enhances fatty acid oxidation in muscles, reduces lipid accumulation, and improves insulin sensitivity. In the intestines, it inhibits cholesterol absorption and promotes lipid excretion. Furthermore, in adipose tissue, curcumin suppresses adipogenesis, stimulates lipolysis, and induces the browning of white adipose tissue. Colored arrows indicate the inhibitory (black) or stimulatory (red) effects of curcumin on downstream signaling targets, finally involved in regulating a variety of intracellular physiological processes. (Original drawing, created with BioRender, https://biorender.com, accessed on 8 January 2026).

The ameliorative effect of curcumin on systemic lipid metabolism is closely linked to its direct regulation of adipocyte function. Its effects are primarily evident in the inhibition of adipogenesis, the promotion of lipolysis and oxidation, and the induction of adipose browning (Figure 4).

Curcumin effectively inhibits adipogenesis and regulates adipocyte differentiation. In animal models, curcumin treatment significantly reduces serum TC, TG, and LDL-C levels in HFD mice while also decreasing epididymal fat weight and adipocyte diameter [93]. Similar studies have demonstrated that curcumin inhibits lipid deposition in the epididymal fat of HFD rats [99], further corroborating its effects at the cellular level by significantly reducing fat weight. At the cellular level, curcumin inhibits the differentiation of mouse 3T3-L1 preadipocytes into mature adipocytes in a dose-dependent manner [106,107]. The underlying mechanisms involve multiple aspects: specifically, curcumin downregulates the expression of fatty acid synthase (FAS), thereby inhibiting the differentiation process and lipid accumulation [107]. It also promotes β-catenin signaling and inhibits miR-17–5p, which enhances Tcf7L2 expression, subsequently synergistically increasing Wnt signaling activity and suppressing adipogenesis [108]. In the epididymal white adipose tissue (eWAT), curcumin inhibits the expression of key enzymes involved in triglyceride synthesis, including GPAT1, ATGL, and DGAT1, which reduces the levels of DAG and its derivatives (such as triacylglycerol (TAG), phosphatidylcholine (PC), and phosphatidylethanolamine (PE)) [109]. Additionally, curcumin downregulates stearoyl-CoA desaturase 1 (SCD1), leading to a decrease in the content of palmitoleic acid and oleic acid. Furthermore, curcumin can promote cholesterol efflux in adipocytes in a dose-dependent manner through the PPARγ-LXRα-ABCA1 axis, which further reduces lipid accumulation [110].

The AMPK signaling pathway plays a crucial role in the regulation of lipid metabolism by curcumin. Research has demonstrated that curcumin decreases intracellular lipid accumulation by activating AMPK activity in 3T3-L1 adipocytes, which inhibits the expression of acetyl-CoA carboxylase (ACC) and reduces the conversion of acetyl-CoA to malonyl-CoA. Additionally, curcumin dose-dependently upregulates carnitine palmitoyltransferase-1 (CPT-1), thereby promoting fatty acid oxidation, while simultaneously downregulating GPAT1 to inhibit fatty acid esterification. This effect has been confirmed in HFD mouse models [106]. Moreover, curcumin treatment significantly lowers the expression of key adipogenic transcription factors, including PPARγ and C/EBPα, in subcutaneous adipose tissue, while concurrently reducing serum cholesterol, TG, free fatty acids, body weight, and body fat content [106]. Mechanistic expansion studies have further elucidated that curcumin inhibits cAMP/PKA signaling through the activation of AMPK, while simultaneously restoring the activity of phosphodiesterase 3B (PDE3B) to enhance cAMP degradation. This dual action synergistically increases AMP levels in adipose tissue and decreases cAMP accumulation, ultimately leading to the inhibition of lipolysis [45]. In the HFD mouse model, curcumin also specifically suppressed the transcriptional activity of SREBP-1c and the activation of hormone-sensitive lipase (HSL) in adipose tissue, resulting in a significant reduction in the release of glycerol and free fatty acids (FFAs) [45].

Furthermore, curcumin promotes the beneficial remodeling of adipocytes, including the induction of white fat browning. Studies conducted by Ding et al. demonstrate that curcumin enhances the expression of uncoupling protein 1 (UCP-1), lipoprotein lipase (LPL), and adiponectin in both brown adipose tissue (BAT) and white adipose tissue (WAT), while simultaneously reducing the expression of fatty acid synthase (FAS), stearoyl-CoA desaturase-1 (SCD-1), and 3-hydroxy-3-methyl-glutaryl-CoA reductase (HMGCR). This modulation contributes to improved fatty acid oxidation metabolism in adipocytes [93].

In summary, curcumin exerts a multidimensional influence on adipocyte function through a regulatory network that encompasses various pathways involved in both fat synthesis and decomposition. This provides a significant mechanistic foundation for the prevention and treatment of obesity and related metabolic diseases (Figure 4).

### 5.4. Curcumin Effect on Muscle Lipid Metabolism

Research has demonstrated that curcumin can significantly alleviate muscle insulin resistance by enhancing lipid oxidation and energy conversion in skeletal muscle. In both diabetic rat skeletal muscle and L6 myotube cells, curcumin activates the LKB1-AMPK signaling pathway, inhibits the activity of acetyl-CoA carboxylase (ACC) to decrease fatty acid synthesis, and upregulates the expression of the fatty acid transporter CD36 and carnitine palmitoyltransferase-1 (CPT-1), thereby promoting the uptake and oxidation of free fatty acids [70]. This effect has also been corroborated in C2C12 mouse skeletal muscle cells [71]. Furthermore, animal studies have shown that curcumin treatment not only significantly increases hepatic glycogen reserves and skeletal muscle lipoprotein lipase (LPL) activity in diabetic mice but also concurrently reduces plasma levels of free fatty acids, cholesterol, and TG [47]. Collectively, this evidence suggests that curcumin is an effective intervention for improving disorders of lipid metabolism in skeletal muscle (Figure 4).

### 5.5. Curcumin Effect on Intestinal Lipid Metabolism

Curcumin’s cholesterol-lowering effect is attributed to its dual inhibition of hepatic synthesis and intestinal absorption, with the latter likely playing a more pivotal role. Due to curcumin’s extremely low bioavailability following oral administration, its high local concentration in the intestine allows it to directly target cholesterol absorption pathways. Specifically, curcumin significantly downregulates the expression of Niemann-Pick C1-Like 1 (NPC1L1), a key transporter involved in intestinal cholesterol absorption, by inhibiting the SREBP-2/HNF1α signaling pathway. This action reduces cholesterol absorption and alleviates hepatic lipid accumulation [102,111,112,113]. This mechanism has been validated in both HFD mice [113,114,115] and ApoE^−/−^ mouse models [116], where curcumin treatment decreases the saturation of bile cholesterol in the intestine, reduces cholesterol absorption in the small intestine, and promotes the excretion of neutral sterols in feces, ultimately leading to a significant reduction in serum and liver cholesterol levels. Therefore, the targeted inhibition of the intestinal cholesterol absorption pathway may represent the primary mechanism through which curcumin exerts its cholesterol-lowering effects (Figure 4).

## 6. Curcumin Modulation of Amino Acid and Protein Metabolism

### 6.1. Multi-Target Regulation of Amino Acid and Protein Metabolism by Curcumin

Amino acids serve not only as the fundamental units of protein biosynthesis but also play a crucial role in regulating various physiological functions. They are involved in hormone synthesis, neurotransmitter production, signal transduction, and energy metabolism [117]. The health and disease states of an organism are fundamentally determined by the dynamic balance within the network of signaling molecules. These molecules maintain physiological homeostasis by precisely regulating cell proliferation, differentiation, and responses to environmental stimuli. An imbalance in their expression directly drives the pathological processes of specific diseases through abnormal signal cascade reactions, leading to cellular dysfunction and tissue-specific damage [117].

Metabolic diseases characterized by complex network dysregulation often pose significant challenges to current therapeutic strategies, which typically focus on single-target interventions. These approaches frequently prove inadequate when addressing the multi-pathway and multi-organ pathological mechanisms inherent in these conditions. In contrast, curcumin, a natural bioactive compound, exhibits multi-target regulatory properties that may offer a more effective therapeutic approach, can extensively modulate the activities of various signaling molecules through multimodal molecular interactions, including covalent modification, hydrophobic interactions, and hydrogen bonding [118]. It exhibits significant potential in regulating amino acid metabolism, modifying protein synthesis, and facilitating catabolism. Preclinical and clinical studies have demonstrated that curcumin significantly influences the levels of branched-chain amino acids (leucine, isoleucine, valine) and functional amino acids (arginine, glycine, alanine) in the body through various metabolic pathways [7,63,119]. Clinical research has also shown that curcumin can notably reduce the levels of branched-chain amino acids and their metabolites, including 3-methyl-2-oxovaleric acid, 3-hydroxyisobutyric acid, and kynurenine, in the serum of patients with obesity or NAFLD [7]. Furthermore, due to its unique chemical structure and molecular conformation, curcumin can directly target and bind to a variety of functional proteins, including inflammatory mediators (such as NF-κB), epigenetic modifying enzymes (such as histone acetyltransferases (HATs) and histone deacetylases (HDACs)), kinases, metabolic enzymes (including glyoxalase I (GLOI) and sarco/endoplasmic reticulum Ca^2+^-ATPase (SERCA)), proteasomes, carrier proteins, and nucleic acid molecules (such as DNA and RNA) [118]. Despite the low bioavailability of curcumin, which limits its therapeutic efficacy, its solubility can be enhanced to some extent by utilizing its binding capacity with carrier proteins. It is important to note that curcumin’s role in amino acid and protein metabolism is not an isolated phenomenon; rather, it exemplifies its systemic and multi-target regulatory capabilities. Extensive research has demonstrated that curcumin significantly influences a variety of functional molecules—such as transcription factors, enzymes, receptors, cell cycle proteins, and inflammatory mediators—through both direct and indirect mechanisms, thereby reshaping cellular signaling networks [7,118,119]. This fundamental characteristic enables curcumin to exert precise integrative regulatory effects in critical biological processes, including amino acid metabolism and protein synthesis and degradation, further underscoring its unique value as a systemic metabolic modulator.

### 6.2. Regulatory Role and Evidence of Curcumin in Muscle Protein Metabolism

Protein serves as the primary component of muscle and is essential for both muscle growth and maintenance. The process of muscle growth is governed by the balance between muscle protein synthesis (MPS) and muscle protein breakdown (MPB), with a direct positive correlation existing between the levels of protein synthesis and muscle mass [120]. Sarcopenia represents a significant pathological factor contributing to frailty and mobility impairments in the elderly population, characterized by a progressive decline in skeletal muscle mass, muscle strength, and physical function [121]. This condition is frequently associated with various diseases, including osteoporosis, obesity, and type 2 diabetes [16,122].

**Figure 5:**
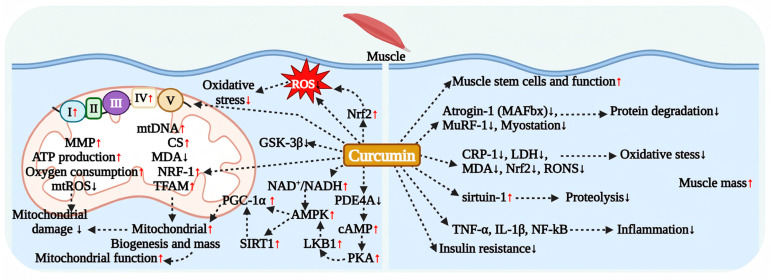
Curcumin Improves Mitochondrial Function in Skeletal Muscle. Curcumin enhances the energy metabolism function of skeletal muscle by activating signaling pathways such as AMPK, promoting mitochondrial biogenesis, and alleviating oxidative stress. Colored arrows indicate the inhibitory (black) or stimulatory (red) effects of curcumin on downstream signaling targets, finally involved in regulating a variety of intracellular physiological processes. (Original drawing, created with BioRender, https://biorender.com, accessed on 8 January 2026).

Recent research has demonstrated that curcumin has considerable potential in enhancing muscle protein metabolism (Figure 5). In a mouse model of diabetic skeletal muscle atrophy, curcumin treatment significantly reduced protein ubiquitination levels in skeletal muscle tissue. It specifically inhibited the expression of ubiquitin E3 ligases Atrogin-1 (also known as MAFbx) and MuRF1 genes, while simultaneously decreasing levels of inflammatory factors such as TNF-α and IL-1β, thereby alleviating oxidative stress. These effects ultimately lead to a significant mitigation of skeletal muscle atrophy symptoms [123]. Additionally, Receno et al. showed in an aged rat model that long-term dietary supplementation with curcumin markedly enhances skeletal muscle mass and motor function [120]. Furthermore, in a mouse model of contusion-induced muscle injury, curcumin effectively increased muscle mass by promoting the synthesis of specific proteins and accelerating the muscle regeneration process [124]. In a rat model of sarcopenia, treatment with curcumin significantly improved muscle endurance, grip strength, and the fat-to-lean mass ratio [125]. Studies examining chronic forced exercise-induced sarcopenia in mice revealed that curcumin treatment markedly increased the weights of the gastrocnemius and soleus muscles, as well as significantly enhanced calf circumference, strength, and total body protein content. This improvement was attributed to the upregulation of genes associated with muscle growth and protein synthesis, alongside the downregulation of genes related to degradation [121]. Research conducted by García et al. confirmed that curcumin effectively mitigated muscle degradation and loss induced by heavy loads in mice by activating the sirtuin-1 (SIRT1) signaling pathway, which in turn attenuated muscle protein hydrolysis and reduced the expression of signaling molecules associated with muscle atrophy [16]. This demonstrates that curcumin precisely integrates amino acid metabolism and protein turnover through its multi-level, multi-target direct and indirect effects, thereby fully revealing its unique value as a systemic metabolic regulator in maintaining protein homeostasis.

### 6.3. Antioxidant and Molecular Mechanisms of Curcumin’s Muscle Protective Effects

The decline of the skeletal muscle oxidative defense system, accompanied by excessive accumulation of reactive oxygen and nitrogen species (RONS) leading to oxidative stress, has been confirmed as a key pathological mechanism responsible for muscle mass loss and motor dysfunction [126] (Figure 5). Antioxidant and mitochondrial protection represent crucial mechanisms underlying curcumin’s muscle-protective effects. Curcumin, a potent antioxidant substantiated by multiple studies, demonstrates multi-target regulatory effects in a sarcopenia model mouse [17]. In a chronic forced exercise-induced sarcopenia model, curcumin treatment significantly alleviated lipid peroxidation, inflammation, and oxidative stress levels in the body while enhancing muscle weight and strength [121]. Treatment with the aqueous extract significantly downregulated the levels of myostatin, MuRF-1, and Atrogin-1, while increasing the activity of muscle antioxidant enzymes and reducing malondialdehyde (MDA) levels, thereby markedly alleviating symptoms of muscle atrophy [127]. Curcumin preserves muscle tissue health by modulating the body’s oxidative stress levels through the activation of the Nrf2 signaling pathway, which serves as a critical regulatory hub for cellular antioxidant defenses [120,128,129]. Furthermore, curcumin indirectly facilitates muscle mass improvement by sustaining the quantity and functional activity of muscle stem cells [130], safeguarding mitochondrial function, and inhibiting chronic low-grade inflammation [131,132,133]. Importantly, insulin, a fundamental hormone in regulating protein metabolism, demonstrates significant anti-proteolytic effects within muscle tissue. The enhancement of the insulin signaling pathway in skeletal muscle by curcumin may further augment its protective effects on muscle mass by promoting protein synthesis pathways [47,70,123].

### 6.4. Clinical Research Evidence

Clinical studies have shown that the curcumin formulation Meriva^®^, whether used alone or in combination with other nutritional supplements, can significantly enhance strength and physical performance in elderly subjects, reducing the risk of sarcopenia [134]. A double-blind, randomized controlled trial evaluated the impact of curcumin supplementation on exercise-induced injuries in moderately active women. The results demonstrated that oral administration of 500 mg/d curcumin for 8 consecutive weeks significantly reduced serum levels of C-reactive protein (CRP), lactate dehydrogenase (LDH), and malondialdehyde (MDA), indicating an improvement in exercise-induced oxidative stress status [135]. Skeletal muscle, recognized as the primary organ for glucose consumption in the body, exhibits a significant negative correlation between its mass and the risk of developing type 2 diabetes. This finding indicates that curcumin therapy, by preserving or enhancing muscle mass, may serve as an important intervention strategy for improving glucose metabolic disorders in patients with T2DM. However, the evidence supporting curcumin’s efficacy primarily derives from in vitro experiments and animal models. Given the positive outcomes demonstrated in existing research, we recommend advancing clinical translational studies to thoroughly investigate the potential benefits of curcumin on human health through systematic human trials.

## 7. Curcumin Regulation of Mitochondrial Respiration

Patients with obesity and related metabolic diseases frequently exhibit oxidative stress and mitochondrial dysfunction in various tissues and organs [136]. The underlying pathology involves defects in mitochondrial biogenesis and damage to the respiratory chain. Mitochondrial biogenesis is the regulatory process through which cells increase both the number and functionality of healthy mitochondria, encompassing proliferation, mtDNA replication, and protein assembly. This process is essential for compensating for functional defects, meeting energy demands, and responding to stress. Cellular respiration, the accompanying energy production process, is primarily governed by the interaction between PGC-1α and NRF1/2, which constitutes the core regulatory pathway. This interaction drives mitochondrial gene transcription and facilitates the synthesis, modification, and repair of mtDNA-related proteins via the activation of mitochondrial transcription factor A (TFAM) [136].

Mitochondria, as the central hub of energy metabolism, serve not only as the primary source of ROS but also as critical targets for their actions [137]. Physiological fluctuations in ROS levels are essential for redox signaling transduction; however, when ROS levels exceed a certain threshold, they can disrupt redox homeostasis, damage respiratory chain complexes, and subsequently trigger mitochondrial metabolic disorders and dysfunction. This cascade of events leads to the accumulation of oxidative damage, ultimately accelerating disease progression [138]. Research indicates that curcumin exerts protective effects through multiple mechanisms, including the regulation of mitochondrial biogenesis, the promotion of mitochondrial dynamic balance (fusion/fission), and the enhancement of metabolic substrate utilization, thereby demonstrating significant potential in the treatment of mitochondrial-related diseases.

### 7.1. Curcumin Regulation of Hepatocyte Mitochondrial Respiration

**Figure 6:**
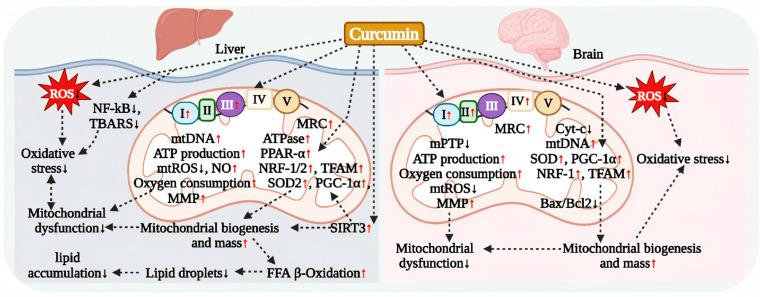
Curcumin improves mitochondrial respiratory function in hepatocytes and neurons. Curcumin enhances mitochondrial biogenesis and respiratory chain function in the liver by upregulating PGC-1α, NRF1, and TFAM. It restores ATP production and reduces oxidative stress, thereby alleviating hepatic steatosis. In neurons, curcumin maintains mitochondrial membrane integrity, enhances antioxidant defense, and protects cells from oxidative stress damage. Colored arrows indicate the inhibitory (black) or stimulatory (red) effects of curcumin on downstream signaling targets, finally involved in regulating a variety of intracellular physiological processes. (Original drawing, created with BioRender, https://biorender.com, accessed on 8 January 2026).

Free fatty acids (FFAs) are primarily transported via the bloodstream to the liver for metabolism, where mitochondria serve as the central site for fatty acid oxidation. In the pathological state of obesity, elevated circulating FFA enhances the liver’s capacity to uptake lipids, thereby directly inducing IR. Concurrently, the resulting hepatic lipid accumulation leads to an overload that triggers abnormal ROS generation. This process, through oxidative stress, impairs mitochondrial biogenesis and induces dysfunction [14,139]. Research indicates that curcumin can ameliorate this pathological process through multiple target mechanisms (Figure 6).

Bing et al. confirmed that in an FFA-induced hepatocyte model, pharmacological concentrations of curcumin significantly enhanced the activities of mitochondrial respiratory chain complexes I and III. This effect reversed the inhibition of mitochondrial respiratory chain (MRC) function and activated the expression of regulatory genes involved in mitochondrial biogenesis, specifically PGC-1α, NRF-1, and TFAM. Additionally, curcumin promoted mtDNA replication, thereby restoring the mitochondrial membrane potential (MMP), increasing ATP levels, and alleviating dysfunction [140]. Further studies demonstrated that in FFA-exposed hepatocytes, curcumin treatment simultaneously reduced intracellular lipid accumulation and mitochondrial ROS levels, restored the mRNA levels of SOD2, NRF1/2, and PGC-1α, and significantly increased mtDNA copy number and ATP production [14]. Notably, this process was accompanied by a reduction in the volume of lipid droplets in hepatocytes, suggesting that curcumin may facilitate metabolic remodeling by enhancing the capacity for fatty acid β-oxidation and improving mitochondrial biogenesis efficiency [14].

In the mouse model of obesity-associated hepatic steatosis (C57BL/6J ob/ob mice), treatment with curcumin significantly restored the function of liver mitochondrial respiratory chain complex I and enhanced ATP production efficiency [96]. This treatment concurrently normalized mitochondrial DNA (mtDNA) copy number and the expression levels of nuclear respiratory factor 1 (NRF1) and mitochondrial transcription factor A (TFAM), while also reducing NF-kB inflammatory signaling and oxidative stress markers such as thiobarbituric acid reactive substances (TBARS) [96]. These effects collectively improved mitochondrial function across multiple dimensions. Furthermore, studies on animal models of chronic kidney disease (5/6 nephrectomy model) have demonstrated that curcumin, by increasing the expression of hepatic mitochondrial PGC-1α and PPARα proteins, not only reverses abnormal mitochondrial membrane potential but also ameliorates mitochondrial metabolic disorders by inhibiting hepatic lipogenesis [141]. In a rat model of overnutrition (postnatal overfed rats), curcumin enhances hepatic mitochondrial biogenesis and the activity of the antioxidant enzyme superoxide dismutase (SOD) by activating the SIRT3 signaling pathway. This activation strengthens the antioxidant defense system and effectively alleviates hepatic steatosis [14]. Furthermore, studies conducted in a diabetic mouse model reveal that curcumin treatment reverses hyperglycemia-induced abnormalities in hepatic lipid oxidation and mitochondrial dysfunction by increasing ATPase activity and restoring the hepatic mitochondrial oxygen consumption rate and nitric oxide (NO) production to normal levels [142]. Collectively, these multi-model evidences demonstrate that curcumin can synergistically reverse the pathophysiology of hepatic steatosis by regulating mitochondrial biogenesis, improving respiratory chain function, and remodeling the redox balance (Figure 6).

### 7.2. Curcumin Regulation of Renal Cell Mitochondrial Respiration

**Figure 7:**
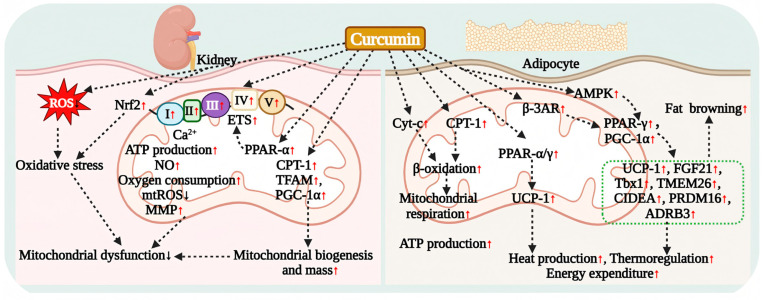
Curcumin improves mitochondrial function in the kidney and adipose tissue. In the kidneys, curcumin enhances oxygen consumption and ATP synthesis efficiency by restoring the activity of mitochondrial respiratory chain complexes (I, II, III, and V). Simultaneously, it promotes mitochondrial biogenesis by upregulating PGC-1α and TFAM, alleviates oxidative stress and endoplasmic reticulum stress, thereby protecting renal function and reducing lipid accumulation and tissue damage. In adipose tissue, curcumin promotes the ‘browning’ of white adipose tissue (WAT), which is characterized by an increase in mitochondrial number and the expression of thermogenic proteins such as UCP1. It upregulates mitochondrial biogenesis and respiratory function by activating signaling pathways such as AMPK/PGC-1α, enhancing energy expenditure, and combating obesity. Colored arrows indicate the inhibitory (black) or stimulatory (red) effects of curcumin on downstream signaling targets, finally involved in regulating a variety of intracellular physiological processes. (Original drawing, created with BioRender, https://biorender.com, accessed on 8 January 2026).

Research indicates that mitochondrial damage is a significant driver of renal inflammation and redox imbalance. In animal models, curcumin has been demonstrated to restore the abnormal respiratory functions of mitochondrial complexes I, II, III, and V, which are induced by renal oxidative stress. This restoration ameliorates mitochondrial dysfunction by enhancing oxygen consumption, ATP production efficiency, Ca^2+^ homeostasis, and transmembrane potential [143]. Similarly, Rojas et al. found that curcumin treatment mitigates mitochondrial dysfunction by stimulating mitochondrial biogenesis, as evidenced by increased levels of PGC-1α and TFAM, thereby reducing renal oxidative damage [144] (Figure 7).

In a diabetic nephropathy model, curcumin significantly reversed renal lipid peroxidation and mitochondrial dysfunction by restoring mitochondrial oxygen consumption and modulating nitric oxide (NO) synthesis. In a mouse model of chronic kidney disease, curcumin treatment partially restored the function of the mitochondrial electron transport system (ETS) by upregulating the expression of PPARα and CPT1, which improved mitochondrial respiration and alleviated β-oxidation impairment. This intervention significantly reduced renal lipid accumulation, mitigated oxidative stress, and alleviated tissue damage [141]. In a mouse model of acute kidney injury, curcumin treatment reduced mitochondrial reactive oxygen species (mtROS) generation and mitochondrial fission through its anti-inflammatory and antioxidant properties, while enhancing mitochondrial biogenesis and ATP synthesis, thereby alleviating mitochondrial damage and exerting protective effects on the kidneys [145].

Additionally, curcumin has been shown to mitigate gentamicin-induced nephrotoxicity in rats by restoring the activities of mitochondrial complexes I and IV, delaying calcium overload-induced mitochondrial permeability transition (MPT), and preventing the collapse of mitochondrial membrane potential (MMP) [146]. Furthermore, cellular experiments indicate that curcumin can inhibit gentamicin-induced renal cell damage by activating the PGC-1α and Nrf2 signaling pathways [146]. These findings suggest that curcumin exerts renal protective effects through a synergistic enhancement of mitochondrial function and promotion of biogenesis (Figure 7).

### 7.3. Curcumin Regulation of Adipocyte Mitochondrial Respiration

Inducing the formation of brown-like adipocytes may serve as a potential strategy for treating obesity and T2DM, primarily through the mechanism of excess energy consumption via thermogenesis. Brown adipose tissue (BAT) exhibits high thermogenic efficiency due to its abundant mitochondrial content, while the key characteristics of white adipose tissue (WAT) browning include an increase in mitochondrial quantity and the ectopic expression of BAT marker proteins.

At the cellular level, curcumin promotes the browning of 3T3-L1 cells and rat primary white adipocytes by activating the AMPK signaling pathway. It upregulates the expression of key browning regulatory factors, such as PPARγ and PGC-1α, as well as brown adipose tissue (BAT) characteristic genes, including UCP1, FGF21, Tbx1, TMEM26, CIDEA, and PRDM16. Furthermore, curcumin significantly increases the number and biogenesis of mitochondria [147]. Additionally, it enhances fatty acid oxidation metabolism in cells by inducing the expression of mitochondrial proteins CPT-1 and cytochrome c in BAT [147] (Figure 7). Zhao et al. reported that curcumin significantly enhances mitochondrial respiratory chain activity and ATP synthesis efficiency by upregulating PPARγ and PGC-1α signaling in 3T3-L1 cells [148]. Notably, curcumin exhibits a concentration-dependent regulation of adipocyte mitochondrial respiration. At low concentrations (10 μM), curcumin markedly improves mitochondrial respiratory function and fuel utilization in 3T3-L1 adipocytes. In contrast, high concentrations (20 μM and 35 μM) inhibit respiratory function due to the disruption of mitochondrial cristae structure and membrane permeability [148].

In animal models, curcumin treatment significantly upregulates the expression of UCP-1, a key thermogenic protein in brown adipose tissue (BAT), through the synergistic activation of peroxisome proliferator-activated receptor alpha (PPARα) and peroxisome proliferator-activated receptor gamma (PPARγ) signaling pathways. This process markedly enhances cold-induced thermogenesis and energy expenditure in HFD mice [149]. Further studies revealed that curcumin also synchronously upregulates the expression levels of PPARγ, peroxisome proliferator-activated receptor gamma coactivator 1-alpha (PGC-1α), and UCP-1 in both white adipose tissue (WAT) and BAT of HFD mice, while inducing the transcriptional activation of PRDM16, a core regulatory molecule involved in WAT browning, thereby significantly improving mitochondrial function in adipocytes [148]. The study conducted by Wang et al. demonstrated that curcumin significantly upregulates the expression levels of PGC-1α and UCP-1, as well as the mitochondrial DNA copy number in the inguinal white adipose tissue (iWAT) of mice, by activating the norepinephrine β3-adrenergic receptor (β3-AR) signaling pathway. Additionally, curcumin promotes the elevated expression of brown adipose tissue (BAT) characteristic genes, including ADRB3, CIDEA, and PRDM16, thereby inducing the transformation of iWAT into a brown-like adipose phenotype [150,151].

Mechanistic studies have demonstrated that curcumin significantly inhibits body weight gain and fat accumulation in HFD mice without altering food intake, while also enhancing their cold tolerance. This effect is likely attributed to the increased activity of brown adipose tissue, the upregulation of thermogenic gene expression in inguinal white adipose tissue (iWAT), and a significant increase in mitochondrial biogenesis, all of which collectively drive the remodeling of adipose tissue energy metabolism [150]. Furthermore, multiple independent studies have corroborated that curcumin markedly improves adipose tissue energy metabolism by upregulating the expression of core thermogenic regulatory factors in adipocytes [152,153,154,155] (Figure 7).

### 7.4. Curcumin Regulation of Muscle Cell Mitochondrial Respiration

Oxidative stress-induced mitochondrial dysfunction in skeletal muscle is a significant contributor to muscle injury [133]. Exercise, as a physiological stimulus, effectively promotes mitochondrial biogenesis in skeletal muscle, thereby enhancing mitochondrial respiratory function. The study conducted by Hamidie et al. demonstrated a synergistic effect between curcumin and exercise intervention. In the gastrocnemius (Gas) and soleus (Sol) muscles of rats, curcumin treatment elevated the NAD^+^/NADH ratio and significantly upregulated indicators related to mitochondrial biogenesis through the activation of the AMPK/SIRT1/PGC-1α signaling pathway. This effect was specifically evidenced by the upregulation of cytochrome c oxidase subunit (COX-IV) protein expression, an increase in mtDNA copy number, enhanced synthesis of OXPHOS subunits, and elevated activity of citrate synthase (CS). The enhancing effect becomes more pronounced when combined with endurance training [131].

Similarly, Ronald et al. reported that curcumin significantly enhances intracellular cAMP levels by increasing the expression of cytochrome c oxidase subunit (COX-IV) and citrate synthase (CS), as well as by improving the activity of mitochondrial complex I. Additionally, curcumin targets the inhibition of phosphodiesterase 4A (PDE4A)-mediated cAMP hydrolysis. This mechanism activates the PKA/LKB-1/AMPK/PGC-1α signaling pathway, systematically enhancing the capacity for mitochondrial biogenesis in the skeletal muscle of exercised rats [156]. These findings indicate that exercise can synergistically amplify the regulatory effects of curcumin on mitochondrial biogenesis (Figure 5).

Under conditions of oxidative stress, curcumin demonstrates distinct regulatory effects on skeletal muscle mitochondria across various pathological models. In the context of HFD-induced metabolic disorders, curcumin specifically reduces the accumulation of ROS and malondialdehyde (MDA) in skeletal muscle mitochondria by activating the Nrf2 signaling pathway, which effectively alleviates muscle oxidative stress [128]. In a chronic kidney disease model, curcumin treatment promotes mitochondrial biogenesis by inhibiting the activity of glycogen synthase kinase 3 beta (GSK-3β) and upregulating the expression of peroxisome proliferator-activated receptor gamma coactivator 1-alpha (PGC-1α), nuclear respiratory factor 1 (NRF-1), and mitochondrial transcription factor A (TFAM). Furthermore, it synergistically restores the activity of mitochondrial respiratory chain complexes and maintains mitochondrial membrane potential homeostasis, thereby effectively mitigating oxidative damage and dysfunction in muscle mitochondria [133].

C2C12 myoblast experiments demonstrated that pharmacological concentrations of curcumin induce moderate ROS production through the induction of NADPH oxidase expression. This process promotes a shift in mitochondrial dynamics towards fusion, which is accompanied by the upregulation of PGC-1α and AMPKα1 protein expression, leading to improvements in mitochondrial quality and the restoration of membrane potential. Consequently, this effectively enhances mitochondrial biological function and alleviates cellular damage induced by heat stress [19]. This phenomenon was further confirmed in animal models, where curcumin treatment significantly ameliorated heat stress-induced pathological damage in skeletal muscle by restoring the structural and functional integrity of mitochondria [19] (Figure 5).

### 7.5. Curcumin Regulation of Neuronal Mitochondrial Respiration

Studies have demonstrated that the distinctive antioxidant properties of curcumin may aid in the prevention or treatment of oxidative stress-induced mitochondrial dysfunction and oxidative damage in neuronal cells [157,158]. This mechanism is associated with curcumin’s capacity to preserve mitochondrial membrane integrity [159], alleviate respiratory dysfunction [33], and promote mitochondrial biogenesis [160] (Figure 6). Research conducted by Stephanie et al. revealed that in vitro, curcumin effectively prevented oxidative stress-induced mitochondrial swelling and dysfunction in brain cells by inhibiting the abnormal opening of the mitochondrial permeability transition pore (mPTP) [161]. Furthermore, animal models have confirmed that curcumin treatment can specifically restore the activity of key enzymes in the mitochondrial respiratory chain across the cerebral cortex, midbrain, and cerebellar regions, including complex I (NADH dehydrogenase), complex II (succinate dehydrogenase), and complex IV (cytochrome oxidase), thereby effectively ameliorating oxidative stress-induced mitochondrial dysfunction [162].

Curcumin exhibits multi-dimensional neuroprotective capabilities in various pathological states of neural injury. At the level of apoptosis regulation, curcumin confers neuroprotection by dynamically maintaining mitochondrial membrane potential and balancing the Bax/Bcl2 ratio, which reduces the efflux of cytochrome c and apoptosis-inducing factors while concurrently decreasing the abnormal accumulation of ROS induced by oxygen-glucose deprivation/reperfusion (OGD/R) [163]. In a mouse model of cerebral ischemia–reperfusion (I/R) injury, curcumin demonstrates neuroprotective effects by activating NRF-1 and TFAM, leading to an increase in mitochondrial number and promoting mitochondrial biogenesis [164]. Additionally, in a rat model of neurotoxicity induced by the heavy metal arsenic, curcumin specifically restores mitochondrial membrane potential in the frontal cortex and hippocampus, while simultaneously enhancing the activity of mitochondrial respiratory chain complexes to exert its neuroprotective effects [33].

The core pathological features of neurodegenerative diseases are intricately linked to mitochondrial dysfunction. Curcumin treatment preserves mitochondrial structural homeostasis by inhibiting Aβ peptide-induced mitochondrial membrane depolarization in neuronal cells. Additionally, it upregulates the expression levels and catalytic activities of superoxide dismutase (SOD) and catalase, thereby effectively ameliorating mitochondrial metabolic defects and the oxidative stress microenvironment [165]. Banji et al. found that curcumin significantly ameliorates oxidative stress-mediated mitochondrial dysfunction in rat brains by enhancing mitochondrial enzyme activity and increasing glutathione biosynthesis levels. This restoration of neuronal biochemical functions effectively reverses cognitive impairment [166]. In the context of aging-related research, curcumin enhances mitochondrial membrane potential and ATP synthesis efficiency by activating the PGC-1α signaling pathway. It also promotes a balance in mitochondrial fusion dynamics, ultimately alleviating mitochondrial dysfunction and delaying the progression of neurodegenerative diseases [167] (Figure 6). These findings provide crucial theoretical support for the translational application of curcumin in treating neurodegenerative disorders.

## 8. Curcumin Regulation of Gut Microbiota and Maintenance of Intestinal Barrier Integrity

### 8.1. Curcumin Regulation of Gut Microbiota via Prebiotic-like Effects

**Figure 8:**
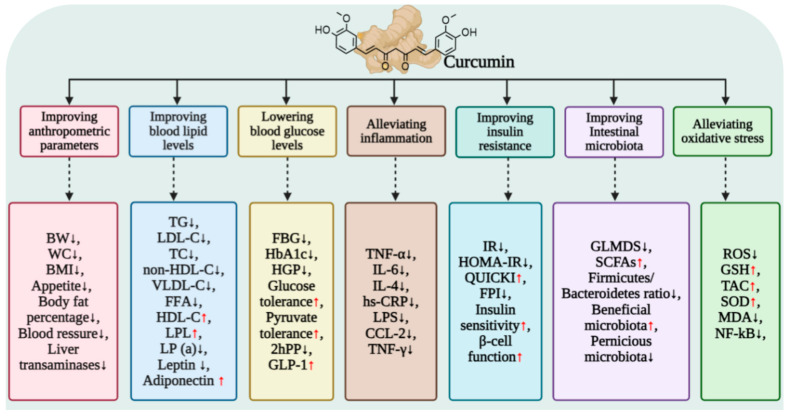
Curcumin improves systemic metabolism and insulin resistance through a multi-organ, multi-mechanism network. Curcumin-mediated systemic metabolic improvement involves a complex multi-organ regulatory network. It integratively ameliorates glucose and lipid metabolism disorders and IR through multiple mechanisms, including reshaping the gut microbiota structure, increasing the production of beneficial metabolites (such as short-chain fatty acids, SCFAs, and bile acids), repairing the intestinal barrier function to reduce endotoxin entry into the bloodstream, and synergistically suppressing systemic inflammation and oxidative stress. Colored arrows indicate the inhibitory (black) or stimulatory (red) effects of curcumin on downstream signaling targets, finally involved in regulating a variety of intracellular physiological processes. (Original drawing, created with BioRender, https://biorender.com, accessed on 8 January 2026).

Research has demonstrated that an imbalance in gut microbiota is closely linked to the progression of various diseases, including obesity, diabetes, cardiovascular diseases, and cancer [168,169]. In contrast, maintaining gut microbiota homeostasis significantly enhances host health [12]. Curcumin, a natural active ingredient commonly utilized in oral medications, is primarily concentrated in the gut and exhibits extremely low systemic bioavailability; however, it manifests a broad spectrum of pharmacological activities. This paradoxical phenomenon indicates that the regulation of gut microbiota may play a crucial role in curcumin’s efficacy. Specifically, curcumin exhibits prebiotic-like properties by modulating the structure of the gut microbiota. It increases the abundance of beneficial bacteria, such as Bifidobacterium, Lactobacillus, and Akkermansia, while simultaneously reducing the load of potential pathogens, including *Coriobacterales*, *Enterobacteria*, *Prevotellaceae*, and *Bacteroidaceae* [170,171]. This modulation optimizes the overall composition and diversity of the microbiota, which subsequently leads to systemic health benefits [7,78]. Therefore, this regulatory effect, based on the “gut-organ axis,” may elucidate curcumin’s intervention in various diseases and offers a novel mechanistic perspective to explain its unique pharmacological phenomenon of “low absorption but high activity” (Figure 8).

### 8.2. Curcumin Ameliorates Obesity and Related Metabolic Diseases

A HFD not only directly contributes to abnormal fat accumulation in the body but also induces metabolic disorders by disrupting the structural diversity of the gut microbiota [172,173]. Studies have demonstrated that gut microbiota dysbiosis and barrier dysfunction are prevalent in obese individuals and animal models, characterized by an abnormal increase in the *Firmicutes*/*Bacteroidetes* (F/B) ratio, which is positively correlated with hepatic steatosis and serves as a biomarker for obesity [168]. Furthermore, epidemiological research has confirmed that gut microbiota dysbiosis is closely associated with the pathological progression of obesity and its complications, including IR, type 2 diabetes, and abnormal glucose and lipid metabolism (Table 1).

Research has confirmed that curcumin exerts a significant protective effect against diet-induced obesity by enhancing intestinal health and effectively alleviating metabolic dysfunction through the core mechanism of regulating intestinal microbiota homeostasis [12]. Multiple studies have demonstrated that in HFD animal models, curcumin treatment not only significantly improves the obesity phenotype but also reshapes the gut microbiota structure by downregulating the F/B ratio, thereby optimizing the composition ratio of beneficial to pathogenic bacteria [12,78,170,174]. Curcumin has been shown to enhance the abundance of beneficial microbiota, specifically *Bacteroidetes*, *Bifidobacterium*, and *Lactobacillus*. Concurrently, it suppresses the proliferation of microbiota linked to metabolic disorders, including *Proteobacteria*, *Desulfovibrio*, and *Prevotellaceae* [170,171,175]. Notably, fecal microbiota transplantation (FMT) experiments have confirmed that transplanting microbiota treated with curcumin into HFD germ-free mice, which lack endogenous intestinal microbiota, can replicate the beneficial metabolic effects of curcumin. This directly demonstrates that the efficacy of curcumin relies on the regulation of intestinal microbiota [49]. Further FMT studies revealed that the microbiota, modulated by curcumin, was enriched with short-chain fatty acid-producing bacteria, such as *Roseburia intestinalis* and *Eubacterium rectale*, which may be key to improving insulin sensitivity [176]. The metabolic improvement effects include reversing the HFD-induced obesity phenotype, alleviating glucose and lipid metabolism disorders (GLMDs), improving IR (evidenced by the downregulation of hepatic gluconeogenesis and de novo lipogenesis (DNL)-related gene expression), restoring pyruvate metabolic balance, and reducing hepatic lipid deposition [49,171].

Clinical studies have further elucidated the direct regulatory effects of curcumin on human gut microbiota. Double-blind randomized trials have demonstrated that curcumin significantly reduces the F/B ratio in patients while increasing the relative abundance of *Bacteroidetes* [40]. Research targeting patients with NAFLD has revealed that curcumin effectively decreases the concentration of harmful metabolites related to gut microbiota, such as endotoxins, in patient serum by specifically modulating the dynamics of gut microbiota. This modulation particularly alters the abundance of specific strains closely associated with disease progression [7]. A randomized placebo-controlled trial has confirmed that curcumin promotes the ecological reconstruction of gut microbiota, playing a critical role in transforming pathogenic bacteria into beneficial bacteria [39]. The aforementioned evidence systematically elucidates the pivotal role of the “microbiota-metabolism” axis in curcumin’s intervention in obesity and related diseases.

### 8.3. Curcumin Enhances Intestinal Barrier Function and Exerts Anti-Inflammatory Effects

An HFD can increase serum lipopolysaccharides (LPS) levels, induce low-grade chronic inflammation, and compromise intestinal barrier function. This impairment allows intestinal endotoxins and bacterial metabolites to leak into the systemic circulation through the damaged intestinal mucosa. These alterations, combined with gut microbiota dysbiosis and damage to the intestinal mucosal barrier, initiate LPS-mediated systemic inflammation, IR, and metabolic endotoxemia, thereby accelerating the progression of obesity and associated metabolic disorders [78,177,178]. Clinical studies have confirmed that dysbiosis of gut microbiota and dysfunction of the intestinal barrier are key triggers driving IR. Repairing the intestinal mucosal barrier, reducing intestinal permeability, and blocking harmful metabolites from entering the bloodstream are considered crucial strategies in the treatment of metabolic diseases. Curcumin exhibits multifaceted regulatory advantages in response to these strategies. Numerous studies have demonstrated that curcumin not only reshapes the gut microbiota structure in HFD and diabetic animal models by increasing beneficial bacteria such as *Akkermansia*, *Bifidobacterium*, and *Lactobacillus* while suppressing endotoxin-producing bacteria like *Enterobacteriaceae* and *Desulfovibrio*, but also enhances intestinal barrier integrity by promoting the expression of ZO-1 and occludin. Additionally, curcumin reduces circulating LPS, inflammatory factors, and uremic toxin levels, thereby effectively ameliorating disorders in glucose and lipid metabolism and reversing IR [49,175,179,180,181,182,183,184]. In the renal disease model, curcumin enhances the intestinal barrier and mitigates kidney damage by promoting beneficial bacteria such as *Lactobacillaceae* and *Ruminococcaceae*, while simultaneously reducing pathogenic bacteria such as *Bacteroidaceae* [10]. Preliminary clinical trials have also confirmed that curcumin treatment can significantly reduce the levels of pro-inflammatory mediators, such as CCL-2, IFN-γ, and IL-4, as well as lipid peroxidation in patients’ plasma. The underlying mechanism may involve the regulation of intestinal microbiota homeostasis [185].

### 8.4. Curcumin Regulation of Bile Acid and Short-Chain Fatty Acid Metabolism

Disruption of bile acid metabolism mediated by gut microbiota is a fundamental mechanism underlying the pathogenesis of obesity, T2DM, and NAFLD [186]. In animal models, curcumin has been demonstrated to enhance bile acid metabolism by remodeling the structure of gut microbiota, significantly ameliorating HFD-induced obesity phenotypes through the enhancement of adaptive thermogenesis and the suppression of weight gain [187]. Clinical trials have further corroborated that curcumin intervention effectively rectifies the gut microbiota-dependent bile acid metabolism imbalance in patients with non-alcoholic simple fatty liver, leading to a significant reduction in hepatic lipid deposition [40]. Short-chain fatty acids (SCFAs), such as acetate, propionate, and butyrate, serve as key substrates for energy metabolism in intestinal epithelial cells and play a crucial role in maintaining the homeostasis of intestinal barrier function and regulating health. Curcumin specifically enhances the abundance of gut microbiota that produce SCFAs, including *Bacteroides*, *Parabacteroides*, *Alistipes*, *Akkermansia*, and *Alloprevotella* [171]. Additionally, it promotes the growth of other SCFA-producing bacteria such as *Roseburia*, *Eubacterium rectale*, *Phascolarctobacterium*, and *Blautia*, resulting in increased SCFA levels in the cecum and colon [176,180]. This regulation effectively restores intestinal barrier function, reduces hepatic steatosis, and inhibits the penetration of intestinal lipopolysaccharides, ultimately reversing obesity and T2DM-related systemic inflammation and IR [10,11,171,180]. Furthermore, beyond its role in metabolic diseases, curcumin can provide protective effects against neurodegenerative diseases through the microbiota-metabolite axis [119,188,189].

Based on the aforementioned evidence, the crucial role of gut microbiota in mediating the protective effects of curcumin metabolism has been adequately established. Importantly, the bidirectional interaction between curcumin and gut microbiota underscores the complexity of its effects, suggesting that the bioactivity of curcumin is influenced not only by the dosage administered but also by individual variations in microbiota composition and metabolic functions; for instance, different microbiota can metabolically convert curcumin into various active metabolites. Concurrently, these metabolites can feed back to regulate the ecological balance of the microbiota. Nevertheless, considering that current clinical data are predominantly derived from small-sample studies, it is essential to clarify the intricate relationship among curcumin, microbiota, and host metabolism through large-scale population studies. This approach would provide evidence-based support for tailored curcumin intervention strategies that account for microbiota characteristics.

## 9. Curcumin Alleviates Inflammation and Oxidative Stress to Improve Systemic Insulin Resistance

As core drivers of IR, the regulation of inflammation and oxidative stress represents a critical mechanism through which curcumin mitigates metabolic disorders. Extensive preclinical and clinical studies have demonstrated that curcumin significantly enhances systemic insulin sensitivity by modulating signaling pathways in adipose, hepatic, and muscle tissues, as well as reshaping the gut microbiota ecosystem (specific mechanisms have been systematically elaborated in previous sections) [190,191]. This section focuses on the evidence of curcumin’s direct actions on anti-inflammatory and antioxidant pathways, elucidating its beneficial effects on improving IR (Figure 8, Table 1).

Chronic low-grade inflammation and oxidative stress are hallmark pathological features observed in high-risk populations for obesity and diabetes, which are further compounded by IR that exacerbates disorders in glucose and lipid metabolism. Characteristic biomarkers of this pathological state include pro-inflammatory factors (TNF-α, IL-1β, IL-6, hs-CRP), anti-inflammatory mediators (IL-10, adiponectin), and indicators of oxidative stress (MDA, GSH, SOD) [192,193,194]. Curcumin has been shown to exert anti-inflammatory and antioxidant effects through multi-target regulation in various cell models and animal experiments. The underlying mechanisms involve the downregulation of pro-inflammatory factors such as TNF-α, IL-6, and resistin, the upregulation of anti-inflammatory adipokines like adiponectin, and the inhibition of NF-kB signaling and MDA generation (specific mechanisms reviewed by Cheng et al.) [195,196,197,198]. Its broad-spectrum anti-inflammatory effects have been further substantiated in clinical studies, encompassing a variety of disease types, including postoperative inflammation [199], chronic anterior uveitis [200], psoriasis [201], inflammatory bowel disease [202], ulcerative colitis [203], vitiligo [204], arthritis [205], pancreatitis [206], and inflammation related to Helicobacter pylori infection [207].

Numerous randomized double-blind placebo-controlled trials have demonstrated that curcumin effectively ameliorates IR and the pathological progression of related metabolic diseases by modulating the inflammation-oxidative stress axis [5,208,209]. This intervention significantly improves metabolic disorders in patients with T2DM. At the pathological mechanism level, curcumin treatment effectively reduces pro-inflammatory markers, including high-sensitivity C-reactive protein (hs-CRP) [20,35,51], IL-6 [20,36], TNF-α [20,36], A-FABP [20], and endothelin-1 [36], while simultaneously increasing adiponectin [35,91] and total antioxidant capacity (TAC) [34,51,210] in patients.

In the context of antioxidant damage, curcumin treatment significantly decreased the levels of oxidative stress products, including malondialdehyde (MDA) [34,36,51,210] and α1-antitrypsin low-density lipoprotein (AT-LDL) [91]. Furthermore, it enhanced the activity of endogenous antioxidant systems, such as superoxide dismutase (SOD) [20,34,51] and glutathione (GSH) [51,210].

The dual regulatory effect of curcumin leads to significant clinical benefits, including enhanced insulin sensitivity, as evidenced by a marked reduction in serum insulin levels and resistin resistance [15], a decreased HOMA-IR index [211], improved β-cell function, and increased adiponectin levels [211]. Additionally, curcumin has been shown to delay the progression from prediabetes to T2DM [211] and to provide sustained metabolic protection even in patients with comorbid coronary heart disease [210]. Notably, novel formulations, such as nano-curcumin, further enhance these beneficial effects by improving curcumin’s bioavailability [36,51]. The clinical evidence supports the conclusion that curcumin can effectively delay the progression of T2DM while simultaneously improving IR through the synergistic regulation of inflammation and oxidative stress signaling. The core mechanism is rooted in the translation of anti-inflammatory and antioxidant effects into the clinical benefits of enhanced insulin sensitivity (Figure 8, Table 1).

**Table 1:** Clinical Evidence of Curcumin in Metabolic Regulation.

Compound	Disease	Dose	Duration	Outcome	Regulatory Ways	Conclusion	Refs
Curcumin	T2DM	1500 mg/d	10 weeks	BW ↓, BMI ↓, WC ↓, FBS ↓	Glycometabolism, Lipid metabolism	Long-term intake of curcumin can effectively improve fasting blood glucose and body weight in patients with T2DM.	[5]
Curcumin	T2DM	300 mg/d	3 months	FBS ↓, HbA1c ↓, HOMA-IR ↓, FFAs ↓, TC ↓, LPL ↑	Glycometabolism, Lipid metabolism	Effectively reduces blood glucose and serum free fatty acids in T2DM patients and promotes fatty acid oxidation.	[52]
Curcumin	T2DM	1500 mg/d	6 months	PWV ↓, adiponectin ↑, leptin ↓, HOMA-IR ↓, TC ↓, uric acid ↓, VF ↓, TBF ↓, LDL-C ↓, TG ↓, HDL-C ↑	Lipid metabolism, Amino acid metabolism, Protein metabolism	Effectively improve atherosclerosis risk and related metabolic disorders in patients with T2DM.	[54]
Curcumin	T2DM	500 mg/d	3 months	FPG ↓, 2hpp ↓, HbA1c ↓, insulin sensitivity ↑, insulin ↓, IR ↓	Glycometabolism	Significantly improve glycemic parameters in T2DM patients.	[6]
Curcumin	NASFL	500 mg/d	24 weeks	weight ↓, BMI ↓, FFAs ↓, TC ↓, FBS ↓, HbA1c ↓, insulin ↓, F/B ratio ↓, Bacteroides ↑, DCA ↑, GLP-1 ↑	Glycometabolism, Lipid metabolism, Amino acid metabolism, Protein metabolism, Intestinal flora, Bile acid metabolism	Reduce liver fat content in NAFLD patients, regulate gut microbiota, and improve bile acid metabolism.	[40]
Curcumin	Obesity	1000 mg/d	30 days	TC ↓	Lipid metabolism	Beneficial for reducing blood lipid levels in obese patients.	[84]
Curcumin	NAFLD	70 mg/d	8 weeks	Liver fat ↓, BMI ↓, TG ↓, HLD-C ↓, TC ↓, AST ↓, ALT ↓, Glucose ↓, HbA1c ↓	Glycometabolism, Lipid metabolism, Amino acid metabolism, Protein metabolism	Short-term curcumin administration significantly improves NAFLD-related pathological features.	[87]
Curcumin	NAFLD	1000 mg/d	8 weeks	TG ↓, HDL-C ↓, TC ↓, non-HDL-C ↓, uric acid ↓	Lipid metabolism, Amino acid metabolism	Significantly reduce blood lipid and uric acid levels in NAFLD patients and improve metabolic profiles.	[88]
Curcumin	NAFLD	50 mg/d	8 weeks	HDL-C ↑, adiponectin ↑, leptin ↓, Leptin/Adiponectin ratio ↓	Lipid metabolism, Protein metabolism	Effectively improve serum adipokine levels in NAFLD patients.	[89]
Curcumin	Healthy people	500 mg/d	8 weeks	CRP ↓, LDH ↓, MDA ↓, VO2 max ↑	Protein metabolism, Redox metabolism, Inflammation	Supplementation of curcumin during moderate-intensity exercise reduces markers of inflammation, oxidative stress, and muscle damage in the body.	[135]
Curcumin	T2MD	1000 mg/d	12 weeks	MDA ↓, TAC ↑, GSH ↑, PPAR-γ ↑	Redox metabolism, Mitochondrial metabolism	Improving inflammation and oxidative stress in patients with T2DM.	[210]
Curcumin	CKD	320 mg/d	8 weeks	Lipid peroxidation ↓, TAC ↑	Redox metabolism	Reduce oxidative stress levels in CKD patients.	[208]
Curcumin	T2DM	1500 mg/d	10 weeks	TG ↓, hs-CRP ↓, adiponectin ↑	Lipid metabolism, Inflammation	Delaying the progression of diabetic complications by reducing triglyceride levels and inflammatory markers.	[35]
Curcumin	T2DM	300 mg/d	3 months	A-FABP ↓, CRP ↓, TNF-α ↓, IL-6 ↓, SOD ↑, Glucose ↓, FFAs ↓	Lipid metabolism, Inflammation, Glycometabolism, Redox metabolism	Reducing serum A-FABP levels in T2DM patients, exerting anti-diabetic effects, and improving metabolic parameters.	[20]
Curcumin	T2DM	1500 mg/d	9 months	β-cell function ↑, HOMA-β ↑, C-peptide ↓, HOMA-IR ↓, adiponectin ↑	Glycometabolism, Lipid metabolism, Protein metabolism	Long-term curcumin intake may delay the progression from prediabetes to T2DM, improve β-cell function, and reduce insulin resistance.	[211]
Nano curcumin	DFU	80 mg/d	12 weeks	FBS ↓, insulin ↓, IR ↓, insulin sensitivity ↑, TG ↓, LDL-C ↓, TAC ↑, GSH ↑	Glycometabolism, Lipid metabolism, Redox metabolism	Improve glucose and lipid metabolism disorders in DFU patients, alleviate insulin resistance, and oxidative stress.	[15]
Nano curcumin	Diabetes on Hemodialysis	80 mg/d	12 weeks	FBS ↓, insulin ↓, TC ↓, VLDL-TG ↓, TG ↓, LDL-TG ↓, TG/HDL-TG ratio ↓, hs-CRP ↓, MDA ↓, TAC ↑, Total nitrite level ↑	Glycometabolism, Lipid metabolism, Mitochondrial metabolism, Redox metabolism	Long-term administration of nano-curcumin can effectively improve the metabolic profile in diabetes on hemodialysis patients.	[51]
Nano curcumin	T2DM	80 mg/d	3 months	HbA1c ↓, FBG ↓, TG ↓, BMI ↓, eAG ↓, LDL-C ↓	Glycometabolism, Lipid metabolism	Reduce serum HbA1C, LDL-C, and BMI levels in patients with T2DM.	[50]
Nano curcumin	NAFLD	80 mg/d	3 months	HDL ↑, QUICKI ↑, nesfatin ↑, WC ↓, FBS ↓, FBI ↓, HbA1c ↓, TG ↓, TC ↓, LDL ↓, HOMA-IR ↓, TNF-α, hs-CRP ↓, IL-6 ↓	Glycometabolism, Lipid metabolism	Effectively improves blood glucose, blood lipids, inflammation, waist circumference, liver enzymes, and the degree of fatty liver in NAFLD patients.	[83]
Phospholipid curcumin	NAFLD	1500 mg/d	8 weeks	TG ↓, LDL-C ↓, HDL-C ↓, TC ↓, non-HDL-C ↓, Lipid profile ↑, AST ↓, ALT ↓, uric acid ↓	Lipid metabolism, Protein metabolism	Alleviate the severity of NAFLD and improve disease progression-related indicators.	[90]
Phospholipid curcumin	NAFLD	250 mg/d	8 weeks	3-methyl-2-oxovaleric acid ↓, 3-hydroxyisobutyric acid ↓, kynurenine ↓, succinate ↓, citrate ↓, α-ketoglutarate ↓, methylamine ↓, trimethylamine ↓, maleate ↓, indophenol sulfate ↓, CDCA ↓, taurocholic acid ↓, lithocholic acid ↓	Amino acid metabolism, Bile acid metabolism, Glycometabolism (TCA Cycle) Intestinal flora	Significantly improve patients’ serum metabolic profiles and exert metabolic regulatory effects.	[7]
Curserin^®^ (phytosomal curcumin)	Obesity	400 mg/d	8 weeks	FPI ↓, HOMA-IR ↓, waistline ↓, blood pressure ↓, TG ↓, HDL-C ↑, transaminase ↓, γ-GT ↓, HSI ↓, Serum cortisol ↓	Glycometabolism, Lipid metabolism, Protein metabolism, Amino acid metabolism	Improving blood glucose, liver function, and serum cortisol levels in overweight patients with impaired fasting glucose.	[85]
Curcumin, Piperine	T2DM	Curcumin (500 mg/d), Piperine (5 mg/d)	3 months	Glucose ↓, C-peptide ↓, HbA1c ↓, ALT ↓, AST ↓	Glycometabolism, Lipid metabolism, Protein metabolism	Improvement of blood glucose and liver-related parameters in T2DM patients through combined use with piperine.	[55]
Curcumin, Piperine	T2DM	Curcumin (1000 mg/d), Piperine (10 mg/d)	12 weeks	TC ↓, non-HDL-C ↓, Lp (a) ↓, HDL-C ↑	Lipid metabolism	Reduce atherosclerotic lipid parameters and lipid levels in T2DM patients, and decrease the risk of cardiovascular diseases.	[86]
Curcumin, Piperine	T2DM	Curcumin (1000 mg/d), Piperine (10 mg/d)	12 weeks	leptin ↓, TNF-α ↓, Leptin/Adiponectin ratio ↓, adiponectin ↑	Inflammation, Lipid metabolism, Protein metabolism	Improving inflammatory factors and adipokine levels in patients with T2DM.	[209]
Curcumin, Piperine	T2DM	Curcumin (1000 mg/d), Piperine (10 mg/d)	8 weeks	TAC ↑, SOD ↑, MDA ↓	Redox metabolism	Significantly improve oxidative stress in T2DM patients.	[34]
Theracurmin^®^ (curcumin preparation)	T2DM	180 mg/d	6 months	AT-LDL ↓, oxidized LDL ↓	Glycometabolism, Lipid metabolism	Reduction of oxidized LDL levels in patients with impaired glucose tolerance or non-insulin-dependent diabetes mellitus.	[91]
Meriva^®^ (curcumin preparation)	CKD	1000 mg/d	6 months	CCL-2 ↓, IFN-γ ↓, IL-4 ↓, Lipid peroxidation ↓, Escherichia-Shigella ↓, Lachnoclostridium ↑, Lactobacillaceae ↑	Inflammation, Redox metabolism, Intestinal flora	Significantly reduce pro-inflammatory mediators and lipid peroxidation levels in CKD patients.	[185]
Meriva^®^ (curcumin preparation)	Healthy older people	500 mg/d	3 months	Exercise capacity parameters (including grip strength, weight lifting, walking, cycling, etc.) and health-related indicators (including proteinuria, oxidative stress level, etc.) were significantly improved.	Protein metabolism, Redox metabolism	Combining a standard diet with moderate exercise helps improve physical strength and bodily functions in the elderly, with potential for preventing sarcopenia.	[134]
NCB-02 (curcumin preparation)	T2DM	150 mg/d	8 weeks	MDA ↓, ET-1 ↓, IL-6 ↓, TNF-α ↓	Inflammation, Redox metabolism	Significantly improves endothelial dysfunction in T2DM patients, reduces inflammatory factors, and oxidative stress levels.	[36]

The upward arrow represents an increase, while the downward arrow represents a decrease.

## 10. Summary and Perspectives

Curcumin exhibits extensive regulatory capabilities within the systemic metabolic network through its multi-target mechanisms of action. These mechanisms include modulation of gut microbiota, enhancement of mitochondrial function, inhibition of inflammation and oxidative stress, and regulation of key signaling pathways, such as AMPK. Its distinctive ‘gut-first’ mode of action and bidirectional interactions with the microbiome effectively elucidate the pharmacological characteristics associated with ‘low absorption yet high activity.’ Current research provides substantial evidence supporting the significant potential of curcumin in ameliorating metabolic disorders, including dysregulation of glucose and lipid metabolism, IR, and NAFLD.

Despite its potential, clinical translation still encounters significant challenges. The quality of clinical evidence in humans requires improvement, individual differences in microbiota can impact the stability of therapeutic efficacy, and there is an urgent need to overcome the technical bottleneck of low bioavailability. Future research should prioritize a deeper mechanistic exploration by employing multi-omics technologies to comprehensively analyze the interaction network of "curcumin-microbiota-host." Additionally, it is essential to promote large-scale, long-term clinical studies to clarify the dose–response relationship and safety of curcumin. Concurrently, establishing biomarker-based precision intervention strategies will facilitate personalized applications, while innovative delivery systems can enhance bioavailability.

Through multidisciplinary integration and deep collaboration among industry, academia, and research institutions, curcumin is anticipated to evolve from a traditional dietary supplement into an evidence-based precision metabolic management solution. This transition offers new avenues for the prevention and treatment of metabolic diseases.

## Data Availability

No new data were created or analyzed in this study. Data sharing is not applicable to this article.

## Abbreviations

TCA	tricarboxylic acid cycle	APO-B	apolipoprotein B
GSK-3β	glycogen synthase kinase-3 beta	APOA-I	apolipoprotein A-I
DAG	diacylglycerol	SRBI	scavenger receptor class B type I
PKC	protein kinase C	HL	hepatic lipase
cAMP	cyclic adenosine monophosphate	ApoE	apolipoprotein E
PKA	protein kinase A	CPT-1	carnitine palmitoyltransferase I
PDE4B	phosphodiesterase 4B	LPL	lipoprotein lipase
PDH	pyruvate dehydrogenase	LKB1	liver Kinase B1
PDK4	pyruvate dehydrogenase kinase 4	VO2 max	maximum oxygen uptake
PC	pyruvate carboxylase	UCP-1	uncoupling protein 1
CREB	cAMP response element-binding protein	TCF7I2	transcription factor 7 like 2
ABCA1	ATP-binding cassette subfamily A member 1	ROS	reactive oxygen species
HK	hexokinase	mtDNA	mitochondrial DNA
PFK2	phosphofructokinase-2	MRC	mitochondrial respiratory chain
GLUT4	glucose transporter 4	SOD2	superoxide dismutase 2
TFAM	mitochondrial transcription factor A	NRF-1/2	nuclear respiratory factor 1/2
PEPCK	phosphoenolpyruvate carboxykinase	SIRT3	sirtuin 3
mPTP	mitochondrial permeability transition pore	Cyt-c	cytochrome c
TGR5	Takeda G protein-coupled receptor 5	NO	nitric oxide
CIDEA	cell death-inducing DFFA-like effector A	ETS	electron transport system
BA	bile acid	β-3AR	beta-3 adrenergic receptor
GPR40/120	G protein-coupled receptor 40/120	Tbx1	t-box transcription factor 1
GS	glutamine synthetase	PRDM16	PR domain containing 16
PLC	phospholipase C	ADRB3	adrenoceptor beta 3
RONS	reactive oxygen and nitrogen species	FGF21	fibroblast growth factor 21
AMPK	AMP-activated protein kinase	TMEM26	transmembrane protein 26
ACC	acetyl-CoA carboxylase	MDA	malondialdehyde
MDH	malate dehydrogenase	SIRT1	sirtuin 1
FFA	free fatty acid	IL-1β	interleukin-1 beta
ACO	acyl-CoA oxidase	BW	body weight
LXRα	liver X receptor alpha	WC	waist circumference
VLDL-C	very low-density lipoprotein cholesterol	BMI	body mass index
PPAR-α	peroxisome proliferator-activated receptor α	LP (a)	lipoprotein (a)
2hPP	2-h postprandial plasma glucose	FBG	fasting blood glucose
ACC1	acetyl-CoA carboxylase 1	HbA1c	hemoglobin A1c
FAS	fatty acid synthase	HGP	hepatic glucose production
SREBP-1	sterol regulatory element-binding protein-1	GLP-1	glucagon-;ike peptide-1
NPC1L1	niemann-pick c1-like 1	IL-6	interleukin-6
HNF-1α	hepatocyte nuclear factor 1 alpha	CCL-2	C-C motif chemokine ligand 2
SREBP2	sterol regulatory element-binding protein-2	TNF-α/γ	tumor necrosis factor-α/γ
FXR	farnesoid X receptor	SCFA	short-chain fatty acid
SLC13A5	solute carrier family 13 member 5	TAC	total Antioxidant capacity
ACAT	acyl-CoA cholesterol acyltransferase	SOD	superoxide dismutase
LDL-C	low-density lipoprotein cholesterol	SE	cholesteryl ester
SCD-1/2	stearoyl-CoA desaturase-1/2	LDLR	low-density lipoprotein receptor
FADS1/2	fatty acid desaturase 1/2	MVK	mevalonate kinase
GPAT1	glycerol-3-phosphate acyltransferase 1	ACLY	ATP citrate lyase
ACS	acyl-CoA synthetase	DNL	de novo lipogenesis
HMGCR	3-hydroxy-3-methylglutaryl-CoA reductase	TG	triglyceride
HMGCS	3-hydroxy-3-methylglutaryl-CoA synthase	TC	total cholesterol
LSS	lanosterol synthase	DHCR24	24-dehydrocholesterol reductase
SC4MOL	sterol-C4-methyl oxidase-like	DHCR7	7-dehydrocholesterol reductase
SS	squalene synthase	FDPS	farnesyl diphosphate synthase
FDFT1	farnesyl-diphosphate farnesyltransferase 1	G6Pase	glucose-6-phosphatase
CYP7A1	cytochrome P450 family 7 subfamily a member 1	DPP4	dipeptidyl peptidase-4
HDL-C	high-density lipoprotein cholesterol	2hpp	2-h postprandial glucose
FBS	fasting blood sugar	DCA	deoxycholic acid
γ-GT	gamma glutamyl transpeptidase	ET-1	Endothelin-1
FPI	fasting plasma insulin	TBF	total body fat
DFU	diabetic foot ulce	PWV	pulse wave velocity
CKD	chronic kidney disease	VF	visceral fat
HSI	hepatic steatosis index	CDCA	chenodeoxycholic acid
